# Accuracy of Machine Learning Algorithms Based on Electroencephalogram in Sleep Apnea Detection: Systematic Review and Meta-Analysis

**DOI:** 10.2196/93378

**Published:** 2026-07-31

**Authors:** Xiangshuo Li, Lulu Wang, Ting Tang, Yuanyuan Chen, Lan Yang, Hao Cai, Chen Wang, Shuxiao Zhang, Ning Ding, Kouying Liu

**Affiliations:** 1 School of Nursing, Nanjing Medical University, 101 Longmian Avenue, Jiangning District, Nanjing, Jiangsu, 211166, China, 86 13851646546; 2 Department of Respiratory and Critical Care Medicine, Jiangsu Province Hospital, Nanjing, Jiangsu, China

**Keywords:** artificial intelligence, machine learning, deep learning, sleep apnea, electroencephalogram, diagnosis

## Abstract

**Background:**

Sleep apnea (SA) is a serious sleep disorder, and its diagnostic gold standard, polysomnography, is costly and time-consuming. Electroencephalogram (EEG) signals, due to their direct correlation with neural activity and ease of extraction, represent a promising tool. Despite increasing research on machine learning (ML) and deep learning for EEG-based SA detection, model performance has not been consistently evaluated.

**Objective:**

This systematic review evaluated the accuracy of ML in detecting SA from EEG data and provided an evidence base for further clinical application and future research.

**Methods:**

Following the PRISMA-DTA (Preferred Reporting Items for Systematic Reviews and Meta-Analyses of Diagnostic Test Accuracy) and PRISMA (Preferred Reporting Items for Systematic Reviews and Meta-Analyses) 2020 expanded checklists, we systematically searched PubMed, Embase, Web of Science, Cochrane Library (CENTRAL), Scopus, IEEE Xplore, and ClinicalTrials.gov databases from inception to April 2026. Studies evaluating the value of ML algorithms for detecting SA based only on EEG data were included. The Quality Assessment of Diagnostic Accuracy Studies-2 and Prediction Model Risk of Bias Assessment Tool for Artificial Intelligence tools were used to assess the risk of bias in each study. Statistical analysis was performed using the *mada* and *metafor* packages in R (version 4.6.0; R Foundation for Statistical Computing) and the Meta-DiSc (version 1.4; Hospital Ramón y Cajal) software. We used GRADE (Grading of Recommendations Assessment, Development and Evaluation) to evaluate the certainty of evidence.

**Results:**

A total of 27 retrospective studies were included. Segment-level analyses showed high diagnostic performance, with a pooled sensitivity of 0.90 (95% CI 0.85‐0.94; 95% prediction interval 0.43‐0.99) and specificity of 0.92 (95% CI 0.87‐0.95; 95% prediction interval 0.46‐0.99). The pooled area under the summary receiver operating characteristic curve was 0.95 (95% CI 0.92‐0.99). Meta-regression identified EEG channel configuration, region, and validation strategy as significant sources of heterogeneity (*P*=.004, *P*=.003, and *P*=.046, respectively). Multichannel EEG, deep learning approaches, and hold-out validation strategies generally demonstrated better diagnostic performance. Only 2 studies evaluated patient-level diagnostic performance, which was summarized qualitatively.

**Conclusions:**

To our knowledge, this is the first systematic review and meta-analysis specifically focused on the diagnostic accuracy of EEG-based ML models in the detection of SA. This meta-analysis indicates that ML models based on EEG demonstrate good diagnostic accuracy in detecting SA at the segment level and show promise as tools for SA screening and clinical decision support. However, most current studies are retrospective segment-level analyses, which may overestimate the practical value of this technology in real-world clinical settings. To reliably integrate EEG-based ML models into clinical diagnostic workflows, further prospective studies incorporating full-night monitoring and patient-level validation are needed.

## Introduction

Sleep apnea (SA) is a serious sleep disorder affecting approximately 936 million people worldwide with moderate to severe cases [1]. Characterized by a reduction in airflow to less than 10% of normal levels during sleep, lasting over 10 seconds, it leads to fragmented sleep and triggers a range of clinical symptoms, including daytime sleepiness, fatigue, and cognitive decline. Recurrent SA events disrupt brain neuroelectric activity patterns, increasing the risk of seizures, stroke, and cardiovascular disease. Specifically, SA was associated with an approximately 2.15-fold higher risk of stroke and a 1.92-fold higher risk of all-cause mortality [2]. According to data from the American Academy of Sleep Medicine, approximately 4% to 19% of adults are affected by SA, while the prevalence can reach as high as 49% among the older population [3]. However, approximately 75% of patients with moderate SA fail to receive timely diagnosis and treatment, imposing a heavy burden on public health [4].

Currently, polysomnography (PSG) serves as the gold standard for diagnosing SA. This assessment involves overnight recording of multiple physiological signals—including electroencephalogram (EEG), electrocardiogram (ECG), and blood oxygen saturation—in a laboratory setting. However, PSG faces limitations such as high costs, lengthy wait times, and dependence on specialized sleep laboratories, which restrict its widespread application [5]. Additionally, manual interpretation of PSG data is time-consuming, labor-intensive, and prone to subjective bias [6]. Therefore, the pursuit of more convenient and efficient auxiliary diagnostic tools holds significant clinical importance.

Researchers attempted to detect SA based on a single biological signal (such as ECG, blood oxygen saturation, or respiratory signals), and the results demonstrated its feasibility [7-9]. EEG, as a noninvasive technique, records the brain’s electrical activity by placing electrodes on the scalp surface [10]. Although EEG has limitations such as the application of electrodes, the use of conductive gel, and potential discomfort associated with prolonged monitoring, compared to other physiological signals such as ECGs and respiratory airflow, EEG is less susceptible to interference from endogenous physiological factors like irregular breathing and arrhythmia when used for sleep monitoring. It directly reflects neuronal activity and sleep states in the brain, providing richer and more direct physiological insights for the assessment of SA [11]. During SA events, EEG signals often exhibit changes in spectral and temporal characteristics associated with sleep stage transitions, arousal responses, or microawakenings. By analyzing features across different frequency bands, SA events can be identified [12]. Recent advances in artificial intelligence (AI) have further accelerated the application of EEG in SA detection. Machine learning (ML) methods can identify discriminative patterns from handcrafted EEG features, whereas deep learning (DL) models enable automatic extraction of complex spatiotemporal representations directly from raw EEG signals through end-to-end learning [13]. In recent years, there has been a steady increase in the number of studies on the automatic detection of obstructive sleep apnea (OSA) using electroencephalography. However, existing studies have primarily focused on algorithm development, exhibiting significant variations in research design and experimental settings. Therefore, a systematic review and meta-analysis is warranted to assess the heterogeneity among these studies and provide comprehensive test performance results. Although there have been reviews discussing the application of ML in SA detection, most studies have not specifically focused on EEG signals [14-16]. In a previous review, Fathima and Ahmed [11] provided a qualitative summary of studies on EEG-based SA detection, but did not conduct a quantitative meta-analysis of diagnostic accuracy. Therefore, there remains a lack of systematic quantitative evidence regarding the overall accuracy of EEG-based ML models in SA detection.

This systematic review aims to enhance understanding of the field by thoroughly analyzing the variations in ML algorithms for detecting SA in EEGs and their impact on diagnostic efficacy, thereby providing a reference for future technological development and clinical translation.

## Methods

### Study Protocol and Registration

This systematic review was conducted in accordance with the PRISMA-DTA (Preferred Reporting Items for Systematic Reviews and Meta-Analyses of Diagnostic Test Accuracy) guidelines, and reporting was supplemented using relevant items from the PRISMA (Preferred Reporting Items for Systematic Reviews and Meta-Analyses) 2020 expanded checklist. For details, see Checklists 1 and 2. The protocol was registered on the PROSPERO website with registration number CRD420251244156. Minor modifications to the statistical analysis plan were made during the review process to improve methodological rigor, including the incorporation of multilevel random-effects models, prediction intervals, and the Hartung-Knapp-Sidik-Jonkman method. These changes did not affect the study objectives, eligibility criteria, or primary outcomes.

### Search Strategy

A comprehensive literature search was developed and conducted in accordance with the PRISMA-S (Preferred Reporting Items for Systematic Reviews and Meta-Analyses Literature Search Extension) guideline [17]. The following databases were systematically searched: PubMed or MEDLINE via the National Library of Medicine, Embase via Elsevier, Web of Science via Clarivate Analytics, Cochrane CENTRAL via Wiley, Scopus via Elsevier, and IEEE Xplore via IEEE. ClinicalTrials.gov was additionally searched to identify ongoing or unpublished studies. The initial search was conducted in October 2025 and updated in April 2026 using the same strategy. The search strategy combined MeSH terms and free-text keywords included “artificial intelligence,” “machine learning,” “deep learning,” “algorithm,” “sleep apnea syndromes,” “electroencephalography,” “EEG,” “diagnostic accuracy,” “sensitivity,” and “specificity.” Boolean operators (AND/OR) were applied, and reference lists of included studies were manually screened. The literature search was conducted independently by the author. No published search filters were used. The selection of studies was carried out independently by 2 researchers (XL and LW); any disagreements were resolved through discussion, and when necessary, a third reviewer (KL) was consulted for a final decision. Detailed search strategies are provided in Multimedia Appendix 1.

### Eligibility Criteria

Studies were eligible for inclusion if they met the following criteria: (1) included adults with SA aged ≥18 years; (2) directly detected SA events using EEG signals alone; (3) explicitly applied either traditional ML or DL algorithms, with specific algorithm types reported; (4) used PSG as the reference standard; and (5) reported sufficient data to directly or indirectly construct 2×2 contingency tables (true positives [TPs], false positives [FPs], true negatives [TNs], and false negatives [FNs]). Studies were excluded if they met any of the following criteria: (1) focused on sleep staging rather than SA detection; (2) did not specifically address SA; (3) were reviews, conference abstracts, case reports, or other nonoriginal research; or (4) lacked sufficient data for analysis or full-text availability.

### Data Collection Process

The data extraction process uses Microsoft Excel spreadsheets, capturing the following information: study ID, publication year, database source, research objective, patient count, internal or external validation, EEG channel names, feature source, cross-validation method, data type, feature extraction method, training set size, test set size, classifier type, algorithm, sensitivity, specificity, accuracy, *F*_1_-score, area under the curve (AUC), TP, TN, FP, and FN. For studies reporting multiple sets of diagnostic performance results, the dataset or model explicitly recommended by the authors and most consistent with the primary objective of the study was preferentially selected for analysis. In cases of missing or unclear data, attempts were made to contact the original authors to obtain the required information. When necessary data were not directly available, 2×2 contingency tables were reconstructed based on reported sensitivity, specificity, and total sample size. Specifically, TPs, FPs, TNs, and FNs were derived using standard formulas for diagnostic test accuracy meta-analysis to enable quantitative synthesis. Data extraction was performed independently by 2 researchers, with discrepancies resolved through discussion or negotiation with a third party.

### Unit of Analysis and Data Partitioning

Studies are categorized into segment-level and patient-level designs based on the unit of analysis. Segment-level studies analyze EEG data using fixed time windows (eg, 30-second epochs and 10-second frames), treating each segment as an independent unit for feature extraction and classification [18]. Patient-level studies aggregate features recorded throughout the night to generate a single diagnostic result for each patient. To maintain sample independence, studies based on subframe analysis typically use nonoverlapping, fixed-length segmentation. However, SA events may span subframe boundaries; some studies use overlapping sliding windows and perform feature aggregation of subframe features at the feature level to generate a single global feature, thereby preserving event integrity while reducing statistical dependencies between samples [19]. At the data partitioning level, the patient-based training-test set division (where all data from a single participant are fully allocated to either the training set or the test set) effectively prevents data leakage and ensures model generalization. Conversely, if EEG segments from the same participant are distributed across both the training and test sets, this may lead to severe information leakage and an overestimation of performance [20].

### Risk of Bias and Applicability

This study used the Quality Assessment of Diagnostic Accuracy Studies-2 (QUADAS-2) tool [21] and the Prediction Model Risk of Bias Assessment Tool for Artificial Intelligence (PROBAST+AI) tool [22] to conduct quality assessments and risk of bias evaluations for the included studies. QUADAS-2 as a quality assessment tool for diagnostic studies, risk of bias and applicability are evaluated across four dimensions: (1) case selection, (2) studies to be evaluated, (3) gold standard, and (4) process and timing. Each dimension is categorized into 3 levels: low risk, high risk, or unclear risk. Given that the included studies used ML algorithms to diagnose SA based on EEG data, we additionally used an AI prediction model to investigate the PROBAST+AI bias risk assessment tool. This tool is an updated version of the Prediction Model Risk of Bias Assessment Tool-2019, specifically designed to evaluate research on predictive models based on regression modeling or AI methods, distinguishing between the model development and model validation phases. The tool assesses risk of bias and applicability across four domains: (1) participants and data sources, (2) predictor variables, (3) outcomes, and (4) analysis. All assessments were independently conducted by 2 reviewers, and disagreements were resolved through discussion or consultation with a third reviewer.

### Data Synthesis

Statistical analysis was performed using the *mada* and *metafor* packages in R (version 4.6.0; R Foundation for Statistical Computing) and the Meta-DiSc (version 1.4; Hospital Ramón y Cajal) software. Spearman correlation coefficients were calculated to assess the presence of threshold effects. When a threshold effect was identified, a summary receiver operating characteristic curve was constructed, and the AUC was reported. Pooled sensitivity, specificity, likelihood ratios, diagnostic odds ratios, and corresponding 95% CIs were synthesized using random-effects models. To address the nonindependence of effect sizes resulting from the reuse of the same publicly available databases across multiple studies and the inclusion of multiple datasets from a single study, a multilevel random-effects model was applied to account for correlations between effect sizes across and within studies, thereby reducing the risk of type I errors arising from violations of the independence assumption [23]. Between-study heterogeneity was assessed using the Cochran *Q* test, the *I*^2^ statistic, and 95% prediction intervals, with prediction intervals used to reflect the potential range of true effects across different study settings and the practical significance of heterogeneity [24]. Between-study variance (τ^2^) was estimated using restricted maximum likelihood, and the Hartung-Knapp-Sidik-Jonkman method [25] was applied to obtain more robust 95% CIs. Subgroup analyses and meta-regression analyses were conducted to further explore the potential sources of heterogeneity. The subgroup variables were prespecified based on clinical relevance and methodological considerations, including EEG channel, region, feature extraction method, validation strategy, classifier category, detection task, and dataset source. Sensitivity analyses were conducted to evaluate the robustness of the pooled results. Publication bias was assessed using the Deeks funnel plot asymmetry test. In addition, the Fagan nomogram was used to evaluate the clinical utility of EEG-based ML models for SA detection.

### Certainty Assessment

The certainty of evidence was assessed using the GRADE (Grading of Recommendations Assessment, Development and Evaluation) framework. The GRADEpro Guideline Development Tool was used to facilitate the assessment. Five domains were considered, including risk of bias, inconsistency, indirectness, imprecision, and publication bias. Certainty was rated as high, moderate, low, or very low according to standard GRADE criteria. Assessments were performed independently by 2 reviewers, with disagreements resolved through discussion and consultation with a third reviewer when necessary.

## Results

### Study Selection

**Figure 1.**
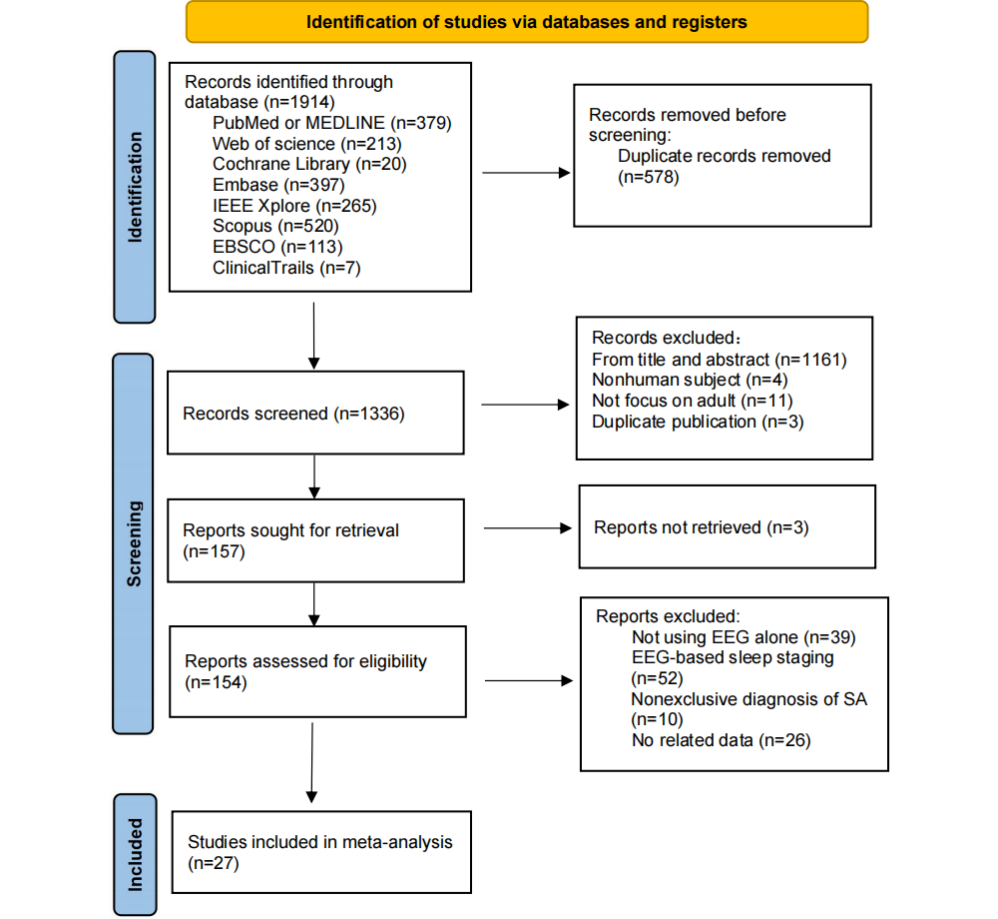
PRISMA (Preferred Reporting Items for Systematic Reviews and Meta-Analyses) flow diagram illustrating the study selection process for a systematic review and meta-analysis of diagnostic test accuracy studies evaluating machine learning algorithms based on EEG signals for the detection of SA. The literature search included multiple electronic databases from inception to April 2026, and records were screened according to predefined eligibility criteria. EEG: electroencephalogram; SA: sleep apnea.

A total of 1914 literature records were identified. After deduplication using EndNote (Clarivate Analytics) software, 578 duplicates were removed. Preliminary screening based on titles and abstracts excluded 1179 studies, leaving 157 studies for full-text eligibility assessment. Following full-text review, 130 studies were excluded, resulting in 27 studies ultimately included in the meta-analysis. The study selection process is illustrated in the PRISMA flow diagram (Figure 1).

### Study Characteristics

The main characteristics of the included studies are summarized in Tables 1 and 2. The studies were published between 2006 and 2026 and were conducted across Asian and Western countries. All 27 studies used retrospective designs. Among them, 3 studies [26-28] performed external validation, while the remaining 24 studies [19,29-51] relied on internal validation. Publicly available datasets were the primary data source, accounting for 80.8% (21/26) of the included studies [19,26-29,31,34-38,41-51]. The most frequently used databases were the MIT-BIH Sleep Database (11/21) and the University College Dublin Sleep Apnea Database (6/21). Notably, the MIT-BIH Sleep Database included exclusively male participants. In addition, 5 studies used self-collected clinical datasets [26,32,33,39,40], and 23 studies reported the specific EEG channels used, with the central region channels being the most frequently used. The proportions of C3-A2 and C4-A1 channels were 47.8% (11/23) [26,28,30,33,34,37-40,45,50] and 56.5% (13/23), respectively [27-29,33,37-41,44,47,49,50]. The included studies applied both ML and DL approaches. Different studies used diverse signal processing and feature extraction techniques, including discrete wavelet transform, variational mode decomposition, fast Fourier transform, Hilbert-Huang transform, and wavelet packet decomposition. These were typically combined to construct multidimensional feature sets incorporating time-domain, frequency-domain, and nonlinear features for model training. Among model evaluation strategies, cross-validation is widely used to obtain more stable assessments of model performance. Specifically, 13 studies used k-fold cross-validation [19,26,29,32,37-40,43,45,49-51], 2 studies used leave-one-out cross-validation [37,47], and 4 studies used the hold-out method to split the data into training and test sets [30,31,33,36]. Detailed information is available in Multimedia Appendix 2 [19,26-51].

**Table 1.** Characteristics of the studies included in this systematic review and meta-analysis of diagnostic test accuracy evaluating machine learning algorithms based on electroencephalogram (EEG) signals for the detection of sleep apnea^a^.

Study	Region	Target	Database	Model type	Algorithms	EEG	Feature extraction	Validation strategy
Cheng et al [26]^b^	Asian countries	Detect OSA^c^	UCDDB^d^, ISRUC^e^, Local hospital	DL^f^	EEG-MIL^g^	C3-A2	Automatic	Cross-validation
Barnes et al [27]^b^	Western countries	Detect SA^h^	SHHS^i^	DL	CNN^j^	C4-A1	Automatic	Cross-validation
Mahmud et al [28]^b^	Asian countries	Detect SA	UCDDB	DL	CNN	C3-A2, C4-A1	Automatic	Independent dataset validation
Jiang et al [29]^b^	Asian countries	Detect SA	MIT-BIH^k^	DL	MSPCNN^l^	O2-A1, C4-A1, C3-O1	Automatic	Cross-validation
Emin Tagluk and Sezgin [30]^b^	Asian countries	Detect OSA	—^m^	ML^n^	ANN^o^	C3-A2	Advanced nonlinear	Hold-out validation
Lin et al [31]^b^	Asian countries	Detect SA	MIT-BIH	ML	ANN	C3-O1	Traditional handcrafted	Hold-out validation
Zhang et al [32]^p^	Asian countries	Screen severe OSA	The Seventh Affiliated Hospital of Sun Yat-sen University	DL	GCN^q^	F3, F4, C3, C4, O1, O2	Advanced nonlinear	Cross-validation
Wang et al [33]^b^	Asian countries	Detect SA	Tianjin Chest Hospital	ML	RF^r^	C3-A2, C4-A1	Traditional handcrafted	Hold-out validation
Prucnal and Polak [34]^b^	Western countries	Detect OSA or CSA^s^	UCDDB	DL	FFNN^t^	C3-A2	Traditional handcrafted	—
Delimayanti et al [35]^b^	Asian countries	Detect SA	CAP Sleep database^u^	DL	CNN	Fp1-F3, F3-C3, C3-P3, P3-O1 and/or Fp2-F4, F4-C4, C4-P4, P4-O2	Automatic	—
Zhou et al [36]^b^	Asian countries	Detect SA	MIT-BIH	ML	SVM^v^	C3-O1	Advanced nonlinear	Hold-out validation
Saha et al [37]^b^	Asian countries	Detect SA	UCDDB	ML	KNN^w^	C3-A2, C4-A1	Traditional handcrafted	Cross-validation (LOOCV)^x^
Gupta et al [38]^b^	Asian countries	Detect SA	MIT-BIH	ML	Ensemble Bagged Trees	C3-A2, C4-A1	Traditional handcrafted	Cross-validation
Wang et al [39]^b^	Asian countries	Detect SA	Tianjin Chest Hospital	DL	BI-LSTM^y^	C3-A2, C4-A1	Automatic	Cross-validation
Zhao et al [40]^b^	Asian countries	Detect OSA or CSA	Tianjin Chest Hospital	ML	RF	C3-A2, C4-A1	Traditional handcrafted	Cross-validation
Bonner et al [41]^b^	Western countries	Detect SA	MIT-BIH	DL	TCNN^z^	C3-O1, C4-A1, O2-A1	—	—
Gurrala et al [42]^b^	Asian countries	Detect SA	MIT-BIH	ML	Ensemble Bagged Tree	—	Traditional handcrafted	—
Taran et al [43]^b^	Asian countries	Detect SA	MIT-BIH	ML	KNN	—	Automatic	Cross-validation
Khan et al [44]^p^	Asian countries	Detect SA	SHHS	ML	SVM	C4-A1	Traditional handcrafted	—
Prucnal and Polak [45]^b^	Western countries	Detect OSA or CSA	UCDDB	ML	SVM	C3-A2	Advanced nonlinear	Cross-validation
Wijaya et al [46]^b^	Asian countries	Detect OSA or CSA	MGH 2018^aa^	DL	CNN-GRU^ab^	C3-M2, C4-M1	Automatic	—
Bhalerao and Pachori [47]^b^	Asian countries	Detect SA	MIT-BIH	DL	CNN	C3-O1, C4-A1, O2-A1	Traditional handcrafted	Cross-validation (LOOCV)
Shahnaz et al [19]^b^	Asian countries	Detect SA	MIT-BIH	ML	SVM	—	Traditional handcrafted	Cross-validation
Taran et al [48]^b^	Asian countries	Detect SA	MIT-BIH	ML	LS-SVM^ac^	—	Advanced nonlinear	—
Sharifi and Fakharzadeh [49]^b^	Asian countries	Detect SA	MIT-BIH	ML	RF	C4-A1	Traditional handcrafted	Cross-validation
Saha et al [50]^b^	Asian countries	Detect SA	UCDDB	ML	KNN	C3-A2, O2-A1, C4-A1, and C3- O1	Traditional handcrafted	Cross-validation
Band and Deshmukh [51]^b^	Asian countries	Detect SA	Sleep EDF Dataset^ad^	DL	1D-CNN^ae^	(FpzCz)	Traditional handcrafted	Cross-validation

a The table summarizes key study characteristics, including study design, geographic region, target population, data sources, model types, machine learning algorithms, EEG channel information, feature extraction methods, and validation strategies.

b Event-level studies.

c OSA: obstructive sleep apnea.

d UCDDB: University College Dublin Sleep Apnea Database.

e ISRUC: Institute of Systems and Robotics, University of Coimbra Sleep Dataset.

f DL: deep learning.

g EEG-MIL: EEG multi-instance learning network.

h SA: sleep apnea.

i SHHS: Sleep Heart Health Study.

j CNN: convolutional neural network.

k MIT-BIH: MIT-BIH Polysomnographic Database.

l MSPCNN: multiscale parallel convolutional neural network.

m Not applicable.

n ML: machine learning.

o ANN: artificial neural network.

p Patient-level study.

q GCN: graph convolutional network.

r RF: random forest.

s CSA: central sleep apnea.

t FFNN: feed-forward neural network.

u CAP Sleep database: Cyclic Alternating Pattern Sleep Database.

v SVM: support vector machine.

w KNN: k-nearest neighbor.

x LOOCV: leave-one-out cross-validation.

y BI-LSTM: bidirectional long short-term memory.

z TCNN: temporal convolutional neural network.

aa MGH 2018: a publicly available dataset derived from polysomnographic recordings collected at Massachusetts General Hospital and released through PhysioNet.

ab GRU: gated recurrent unit.

ac LS-SVM: least squares support vector machine.

ad Sleep EDF Dataset: Sleep European Data Format Database.

ae 1D-CNN: one-dimensional convolutional neural network.

**Table 2.** Data extracted from the included studies^a^.

Study	Sample size (subjects/segments), n	Sensitivity (%)	Specificity (%)	Accuracy (%)
Cheng et al [26]	25/9877	80.35	67.21	70.76
Cheng et al [26]	61/23,728	76.41	62.5	64.9
Cheng et al [26]	35/13,171	82.19	70.30	74.10
Barnes et al [27]	2691/30,087	81.62	67.56	69.92
Mahmud et al [28]	12/287,437	90.06	82.94	86.38
Jiang et al [29]	16/2640	93.08	83.90	89.09
Emin Tagluk and Sezgin [30]	20/4700	94.13	98.17	96.15
Lin et al [31]	N/A^b^/283	69.64	44.44	54.42
Zhang et al [32]	88/N/A	80.77	83.87	82.95
Wang et al [33]	30/406	93.10	95.07	94.33
Prucnal and Polak [34]	N/A/198	86.36	83.33	88.76
Prucnal and Polak [34]	N/A/198	74.24	87.88	89.63
Delimayanti et al [35]	5/12	100.00	83.30	92.00
Zhou et al [36]	12/144	93.2	98.60	95.10
Saha et al [37]	5/1706	89.68	91.79	90.74
Gupta et al [38]	5/1706	93.20	97.20	95.10
Wang et al [39]	N/A/1390	88.46	90.07	89.14
Zhao et al [40]	30/347	90.24	87.95	84.34
Zhao et al [40]	30/347	68.29	96.23	83.33
Bonner et al [41]	15/1219	55.46	91.21	84.50
Gurrala et al [42]	18/9401	94.19	98.73	97.69
Taran et al [43]	N/A/2142	95.68	96.22	96.00
Khan et al [44]	547/N/A	56.82	63.41	60.15
Prucnal and Polak [45]	25/4119	62.52	81.10	74.92
Prucnal and Polak [45]	25/4119	62.20	81.25	74.90
Wijaya et al [46]	N/A/5169	98.95	99.79	99.52
Wijaya et al [46]	N/A/5169	99.36	99.62	99.54
Bhalerao and Pachori [47]	14/7998	97.58	96.72	96.74
Shahnaz et al [19]	14/2720	88.52	85.14	86.84
Taran et al [48]	16/2124	100.00	91.99	96.46
Sharifi and Fakharzadeh [49]	N/A/3640	85.19	89.16	87.18
Saha et al [50]	16/3000	80.97	80.13	80.55
Saha et al [50]	25/4700	91.57	85.19	88.38
Band and Deshmukh [51]	N/A/400	92.50	86.00	89.25

a The table presents the included studies, sample sizes (subjects/segments), and reported diagnostic performance metrics, including sensitivity, specificity, and accuracy. Multiple rows within the same study represent different datasets or distinct diagnostic objectives reported in the original paper.

b N/A: not applicable.

### Risk of Bias and Applicability

**Figure 2.**
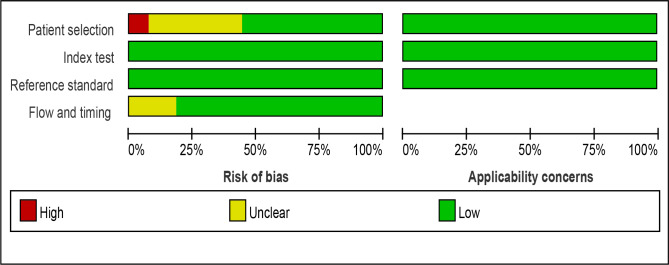
Risk of bias and applicability assessment of included studies using the QUADAS-2 tool in this systematic review and meta-analysis of diagnostic test accuracy evaluating machine learning algorithms based on electroencephalogram signals for sleep apnea detection. The figure summarizes domain-level judgments of risk of bias and applicability concerns. Green, yellow, and red indicate low, unclear, and high risk of bias, respectively. QUADAS-2: Quality Assessment of Diagnostic Accuracy Studies-2.

**Figure 3.**
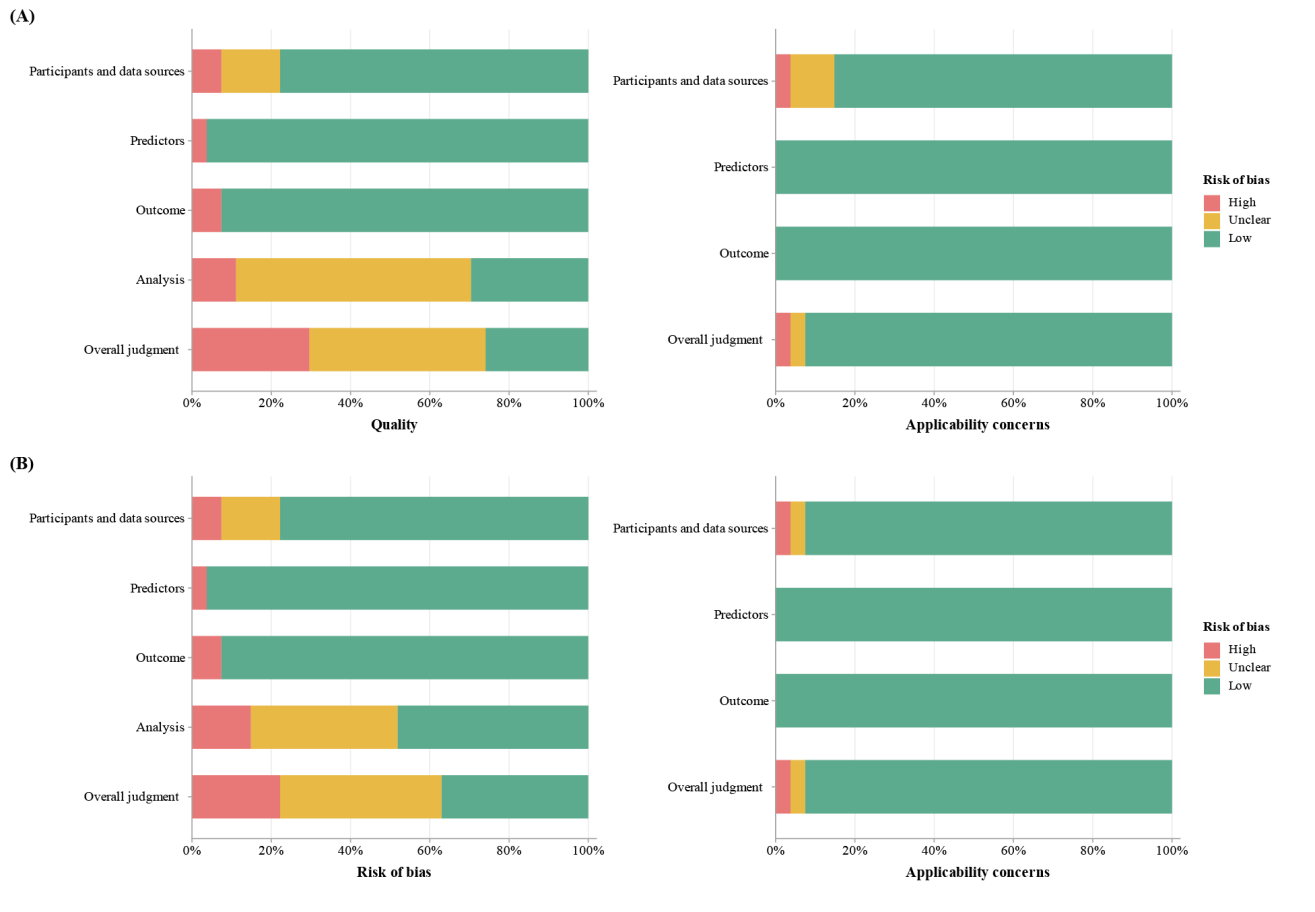
Methodological quality and risk of bias assessment of included studies using the PROBAST+AI tool in this systematic review and meta-analysis of diagnostic test accuracy evaluating machine learning models based on electroencephalogram signals for sleep apnea detection. (A) The assessment results for model development studies. (B) Results for external validation studies. Green, yellow, and red indicate low, unclear, and high risk of bias, respectively. PROBAST+AI: Prediction Model Risk of Bias Assessment Tool for Artificial Intelligence.

Quality assessments of included studies were conducted using the QUADAS-2 and PROBAST+AI tools (Figures 2 and 3). The QUADAS-2 assessment results showed that, in the area of patient selection, 2 (7.4%) studies were judged to be at high risk of bias due to inappropriate subject selection and artificial balancing of groups. Another 10 (37.0%) studies did not adequately describe the subject selection process, and their risk of bias was judged to be unclear; the remaining 15 (55.6%) studies were classified as having a low risk of bias. In the areas of flow and timing, the interval between the test under evaluation and the reference standard was unclear in 5 (18.5%) studies, and the risk of bias was judged to be unclear. All other areas were classified as having a low risk of bias. According to the PROBAST+AI assessment criteria, during the model development phase, 8 (29.6%) studies had a high risk of bias, and 1 (3.7%) study had significant concerns regarding clinical applicability. During the model validation phase, 6 (22.2%) studies were assessed as having a high risk of bias, and 1 (3.7%) study had significant issues regarding clinical applicability. The analysis domain was the primary source of overall bias risk; most studies did not address potential overfitting issues or did not specify how missing data were handled. Overall, the majority of the included studies had a low to moderate risk of bias.

### Synthesis of Results

**Figure 4.**
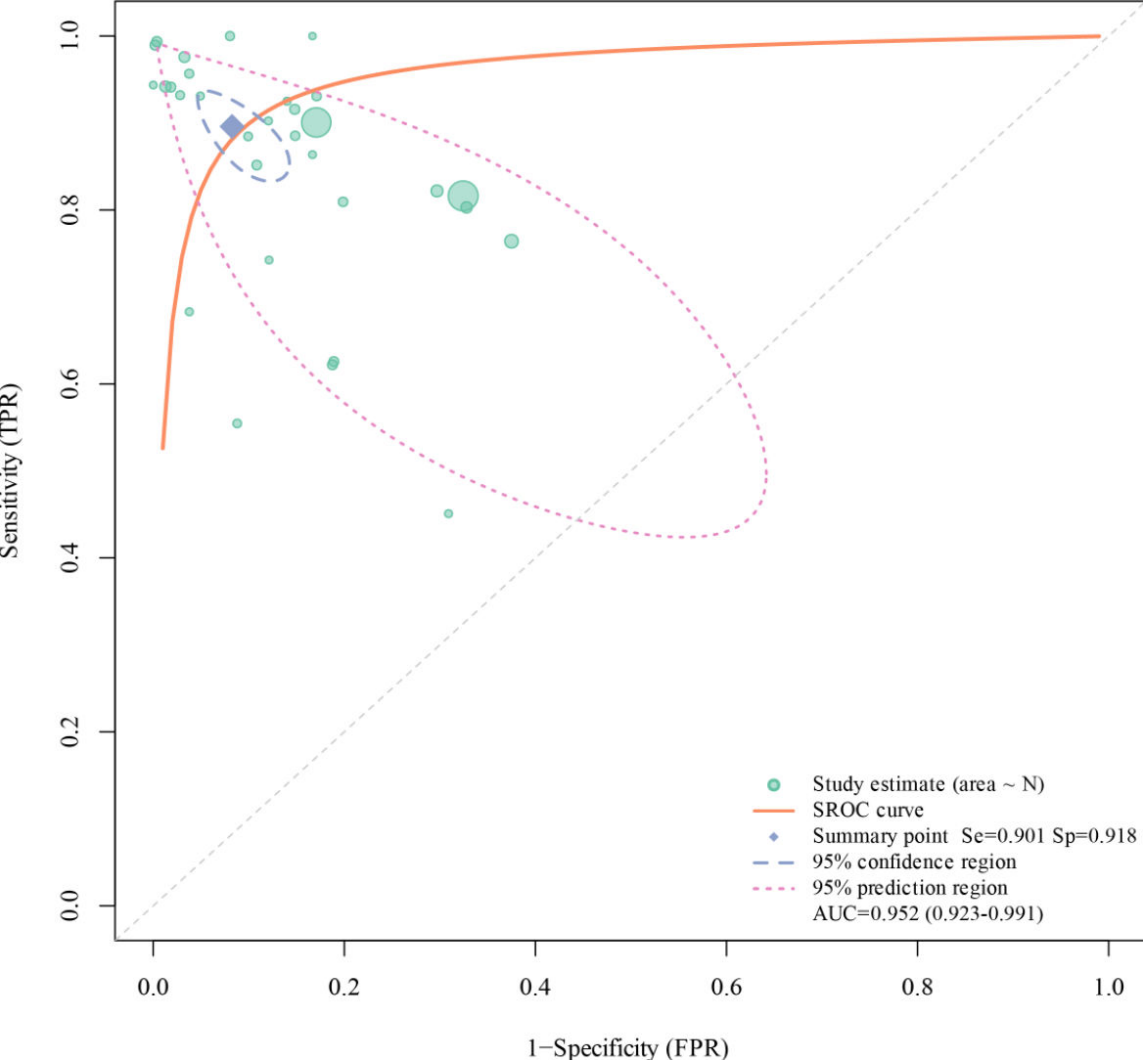
The SROC graph for the studies. The AUC of electroencephalogram-based machine learning algorithms for detecting sleep apnea was 0.95 (95% CI 0.92‐0.99). AUC: area under the curve; FPR: false positive rate; Se: sensitivity; Sp: specificity; SROC: summary receiver operating characteristic; TPR: true positive rate.

**Figure 5.**
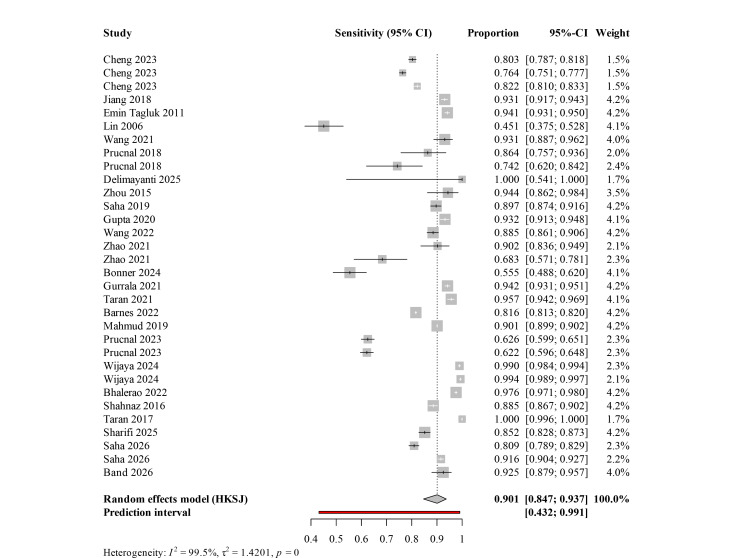
Forest plot of pooled sensitivity for segment-level machine learning and deep learning models. The pooled sensitivity was 0.90 (95% CI 0.85‐0.94), with a 95% prediction interval of 0.43‐0.99 [19,26-31,33-43,45-51]. HKSJ: Hartung-Knapp-Sidik-Jonkman.

**Figure 6.**
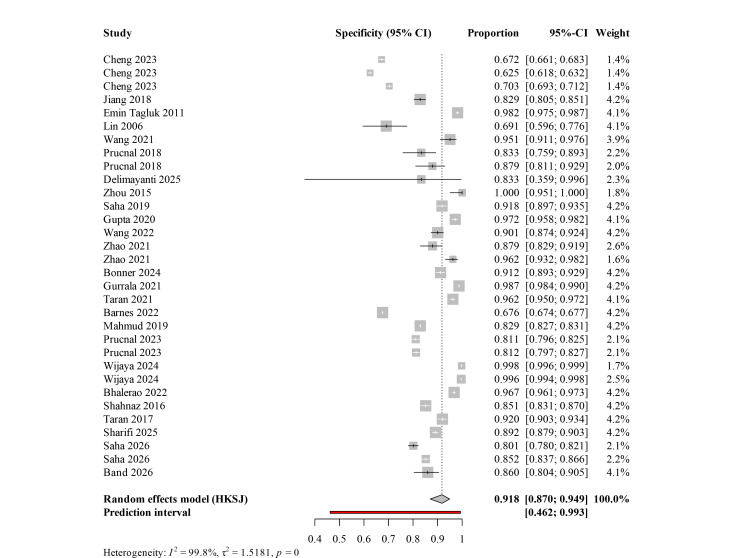
Forest plot of pooled specificity for segment-level machine learning and deep learning models. The pooled specificity was 0.92 (95% CI 0.87‐0.95), with a 95% prediction interval of 0.46‐0.99 [19,26-31,33-43,45-51]. HKSJ: Hartung-Knapp-Sidik-Jonkman.

**Figure 7.**
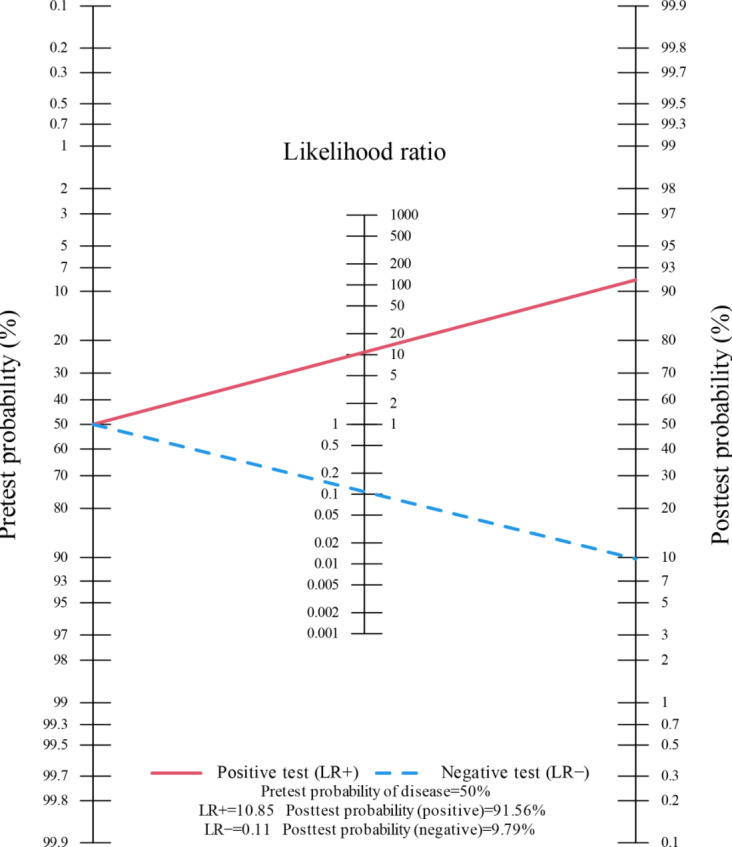
Fagan nomogram of machine learning or deep learning models for the diagnosis of sleep apnea. The first column of the nomogram represents pretest probability, the second column represents likelihood ratio, and the third column shows posttest probability. LR+: positive likelihood ratio; LR−: negative likelihood ratio.

**Figure 8.**
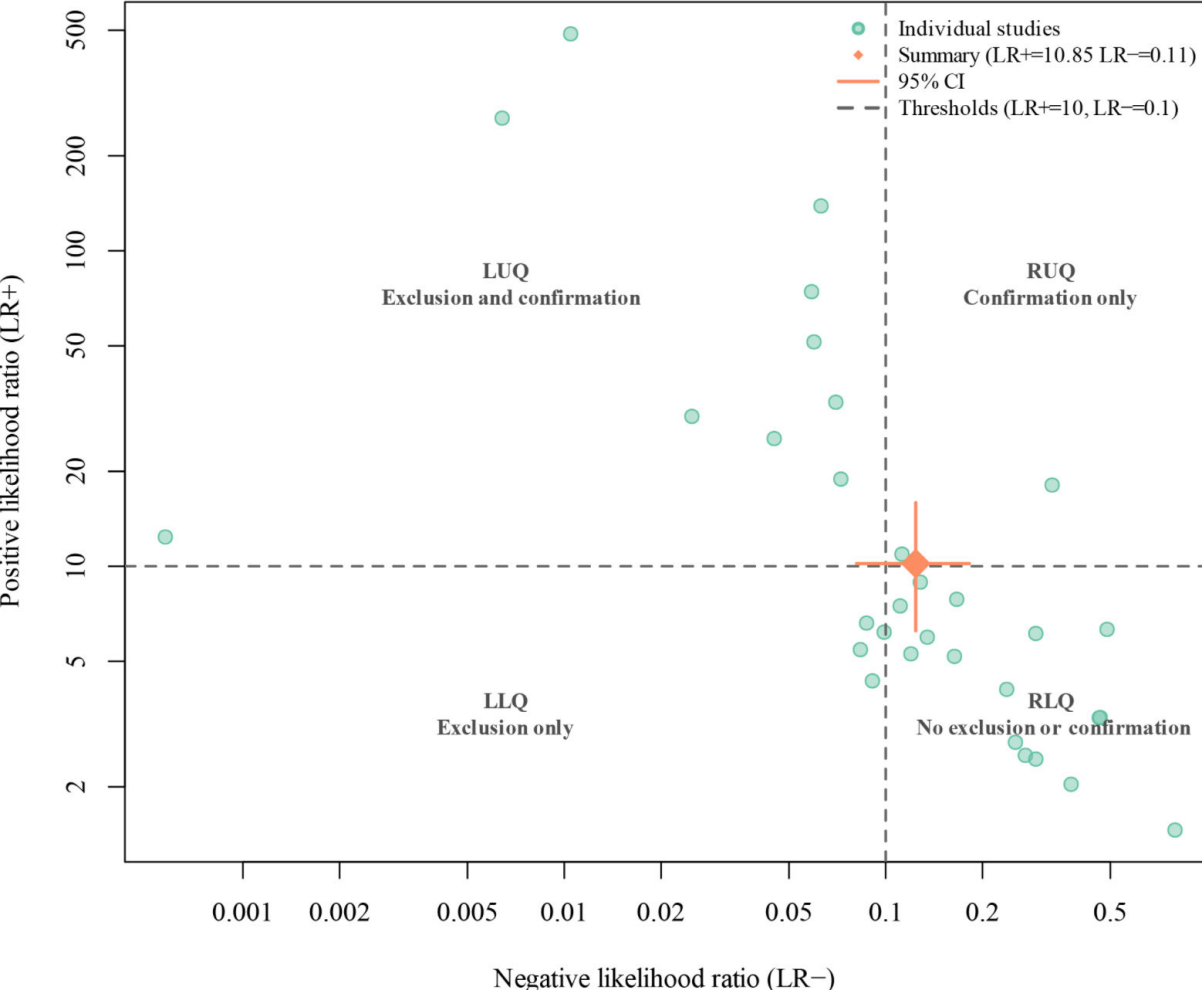
Likelihood ratio scatter plot of electroencephalogram-based machine learning or deep learning diagnostic models. The summary point for machine learning or deep learning models is in the lower-right quadrant (LR+<10 and LR−>0.1; cannot rule out or confirm sleep apnea). LLQ: lower left quadrant; LR+: positive likelihood ratio; LR−: negative likelihood ratio; LUQ: left upper quadrant; RLQ: right lower quadrant; RUQ: right upper quadrant.

A segment-level meta-analysis included 27 studies comprising 32 sets of 4-cell table data. Spearman correlation analysis demonstrated a significant correlation between logit-transformed sensitivity and logit(1−specificity) (*r*=−0.685; *P*<.001), indicating a pronounced threshold effect among the included studies and suggesting that between-study heterogeneity was primarily attributable to differences in classification thresholds. Therefore, a bivariate random-effects model was used to construct the summary receiver operating characteristic curve. The pooled AUC was 0.95 (95% CI 0.92‐0.99; Figure 4), showing a promising overall diagnostic performance of EEG-based ML models for SA detection. The pooled sensitivity was 0.90 (95% CI 0.85‐0.94; *I*^2^=99.5%; Figure 5), and the pooled specificity was 0.92 (95% CI 0.87‐0.95; *I*^2^=99.8%; Figure 6). The diagnostic odds ratio was 88.58 (95% CI 35.76‐219.41). Although the pooled estimates suggested high diagnostic accuracy for both positive and negative SA cases, substantial between-study heterogeneity was observed, indicating considerable variability in model performance across different study settings. Furthermore, the 95% prediction intervals were wide for both sensitivity (0.43‐0.99) and specificity (0.46‐0.99), suggesting that diagnostic performance may vary substantially in external validation scenarios or future real-world applications, thereby reflecting limited model stability and generalizability. To further evaluate potential clinical utility, Fagan nomograms were constructed. When the pretest probability was set at 50%, the positive likelihood ratio was 10.85 (95% CI 6.39‐17.83), corresponding to a posttest probability of 91%, whereas the negative likelihood ratio was 0.11 (95% CI 0.07‐0.17), corresponding to a posttest probability of 11% (Figure 7). However, the pooled likelihood ratio scatterplot showed that most studies were located in the lower-right quadrant (positive likelihood ratio: <10 and negative likelihood ratio: >0.1), indicating limited ability of these models to independently confirm or exclude SA (Figure 8). In addition, substantial dispersion across studies further suggested considerable variability in diagnostic performance. Sensitivity analyses demonstrated that sequential exclusion of individual studies resulted in changes of less than 5 percentage points in pooled sensitivity and specificity estimates, indicating good robustness and stability of the pooled findings (Multimedia Appendix 3) [19,26-31,33-43,45-51].

Among patient-level SA detection studies, only 2 studies met the inclusion criteria. Due to the limited number of eligible studies, reliable estimation of pooled effect sizes was not feasible, precluding formal meta-analysis. Descriptive analysis demonstrated substantial variability in diagnostic performance, with sensitivity ranging from 56.82% to 80.77% and specificity ranging from 63.41% to 83.87%.

### Additional Analysis

At the segment level, this study investigated potential sources of heterogeneity across studies through subgroup analysis and meta-regression. The results of the subgroup analysis showed that only the pooled sensitivity differences among the region subgroups were statistically significant (*P*=.01), while the differences in pooled sensitivity and specificity for the remaining subgroups were not statistically significant (sensitivity: *P*=.94, *P*=.70, *P*=.11, *P*=.83, and *P*=.88, respectively; specificity: *P*=.46, *P*=.71, *P*=.12, *P*=.23, and *P*=.35, respectively). Furthermore, this study originally planned to further evaluate the impact of different detection tasks on diagnostic performance; however, due to the limited number of studies in some subgroups and unstable model convergence, reliable subgroup estimates could not be obtained, and therefore, the results of this analysis are not reported. Detailed subgroup analysis results are presented in Tables 3 and 4. To further identify sources of heterogeneity, this study conducted a meta-regression analysis using prespecified covariates. The results showed that the number of EEG channels, region, and validation method were all significantly associated with pooled diagnostic performance (*P*=.004, *P*=.003, and *P*=.046, respectively), suggesting that differences in EEG channel configuration, region, and validation strategies across studies may represent important sources of heterogeneity and may partially explain the variability among study results. Detailed results are provided in Multimedia Appendix 3.

**Table 3.** The diagnostic performance of electroencephalogram (EEG)-based machine learning (ML) models across different subgroups, including sensitivity and 95% CI^a^.

Category	Studies, n	Sensitivity (%)	95% CI (%)	*I*^2^ (%)	τ^2^	*Q* ^b^	Test for subgroup differences, *P* value
Region							.01
Asian	26	0.92	0.88‐0.95	98.9	1.2362	2224.83	
Western	6	0.71	0.52‐0.85	99.2	0.3792	659.56	
Database							.94
Publicly available	26	0.90	0.83‐0.94	99.5	1.6981	5489.33	
Self-collected	5	0.86	0.72‐0.93	91.7	0.4275	48.42	
Feature extraction							.70
Automatic learning	12	0.90	0.78‐0.96	99.7	1.6716	3577.17	
Traditional handcrafted	15	0.88	0.81‐0.93	98.1	0.8644	719.98	
EEG							.11
Single channel	21	0.87	0.79‐0.93	98.9	1.3708	1770.51	
Multichannel	10	0.94	0.86‐0.98	98.0	1.3645	443.04	
Validation method							.83
Cross-validation	19	0.89	0.83‐0.92	99.0	0.6968	1747.14	
Hold-out validation	4	0.88	0.41‐0.99	99.0	2.1475	290.33	
Algorithm							.88
ML	17	0.90	0.82‐0.94	99.0	1.3537	1614.25	
DL^c^	15	0.90	0.80‐0.96	99.6	1.5616	3840.28	

a Subgroups encompass region, diverse data sources, feature extraction, EEG channel configurations, validation methods, and algorithm types. *P* values indicate the statistical significance of differences in sensitivity between subgroups. Subgroup counts represent independent effect sizes rather than the number of included studies.

b *P* value<.001.

c DL: deep learning.

**Table 4.** The diagnostic performance of electroencephalogram (EEG)-based machine learning (ML) models across different subgroups, including specificity and 95% CI^a^.

Category	Studies, n	Specificity (%)	95% CI (%)	*I*^2^ (%)	τ^2^	*Q* ^b^	Test for subgroup differences*, P* value
Region							.15
Asian	26	0.93	0.88‐0.96	99.7	1.6236	8195.2	
Western	6	0.83	0.67‐0.92	99.3	0.4462	674.15	
Database							.46
Publicly available	26	0.91	0.85‐0.95	99.8	1.6758	15,506.47	
Self-collected	5	0.90	0.74‐0.97	98.1	0.8351	210.64	
Feature extraction							.71
Automatic learning	12	0.90	0.74‐0.97	99.9	2.4597	12,331.54	
Traditional handcrafted	15	0.92	0.86‐0.95	98.5	0.9649	964.99	
EEG							.12
Single channel	21	0.90	0.83‐0.94	99.5	1.2399	4211.78	
Multichannel	10	0.92	0.87‐0.99	99.0	1.9297	938.56	
Validation method							.23
Cross-validation	19	0.89	0.83‐0.93	99.5	0.7881	3697.45	
Hold-out validation	4	0.95	0.55‐1.00	98.1	2.6500	154.79	
Algorithm							.35
ML	17	0.93	0.88‐0.96	98.7	1.1067	1218.63	
DL^c^	15	0.90	0.77‐0.96	99.9	2.0503	12,901	

a Subgroups encompass region, diverse data sources, feature extraction, EEG channel configurations, validation methods, and algorithm types. *P* values indicate the statistical significance of differences in sensitivity between subgroups.

b *P* value<.001.

c DL: deep learning.

### Publication Bias

**Figure 9.**
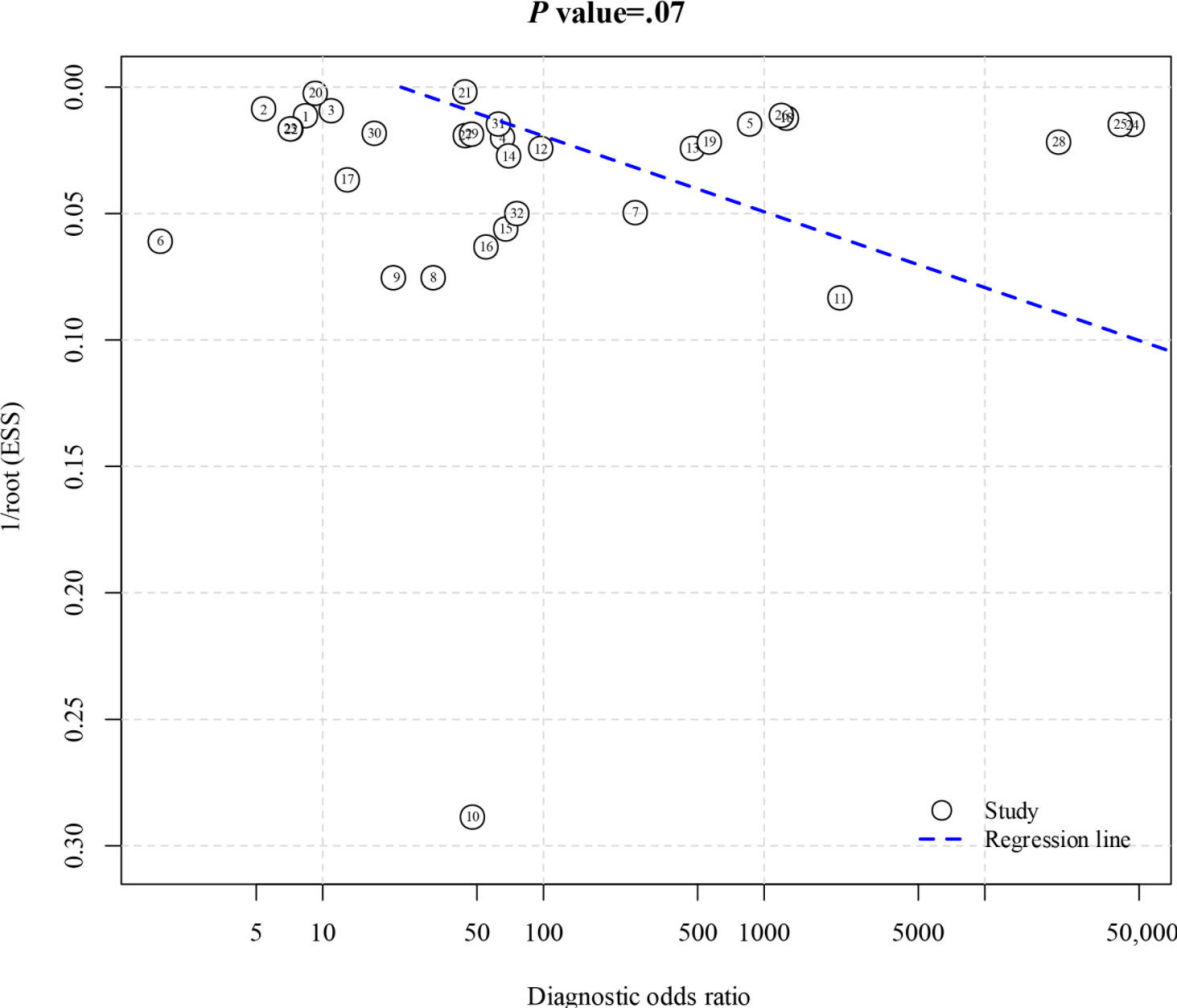
Deeks funnel plot asymmetry test for publication bias in this systematic review and meta-analysis of diagnostic test accuracy evaluating electroencephalogram-based machine learning algorithms for sleep apnea detection. The *P* value of the asymmetry test was .07 (*P*>.05), suggesting no evidence of significant small-study effects. ESS: effective sample size.

Publication bias was primarily assessed using the Deeks funnel plot asymmetry test, which is the recommended method for meta-analyses of diagnostic test accuracy [52]. The results indicate that studies included based on segment-level hierarchy were distributed on both sides of the regression line (*P*=.07), suggesting no evidence of significant small-study effects (Figure 9).

### Certainty of Evidence

According to the GRADE framework, the quality of evidence for both the sensitivity and specificity of this measure was rated as low. However, because several included studies were at high risk of bias and substantial residual heterogeneity remained unexplained after subgroup analyses and meta-regression, the evidence grade was downgraded by 2 levels (Table 5).

**Table 5.** GRADE^a^ assessment of the certainty of evidence for the diagnostic accuracy of electroencephalogram-based machine learning models in detecting sleep apnea^b^.

Outcome	Studies, n	Patients, n	Study design	Factors that may decrease certainty of evidence					Effect per 1000 patients tested			Test accuracy CoE^c^
				Risk of bias	Indirectness	Inconsistency	Imprecision	Publication bias	Pretest probability of 15%, n (95% CI)	Pretest probability of 30%, n (95% CI)	Pretest probability of 50%, n (95% CI)	
True positives	27	705,115	Cross-sectional (cohort-type accuracy study)	Serious^d^	Not serious	Serious^e^	Not serious	None	135 (128-141)	270 (255-282)	450 (425-470)	⨁⨁◯◯ Low
False negatives	27	705,115	Cross-sectional (cohort-type accuracy study)	Serious^d^	Not serious	Serious^e^	Not serious	None	15 (9-22)	30 (18-45)	50 (30-75)	⨁⨁◯◯ Low
True negatives	27	705,115	Cross-sectional (cohort-type accuracy study)	Serious^d^	Not serious	Serious^e^	Not serious	None	782 (739-808)	644 (609-665)	460 (435-475)	⨁⨁◯◯ Low
False positives	27	705,115	Cross-sectional (cohort-type accuracy study)	Serious^d^	Not serious	Serious^e^	Not serious	None	68 (42-111)	56 (35-91)	40 (25-65)	⨁⨁◯◯ Low

a GRADE: Grading of Recommendations Assessment, Development and Evaluation.

b A comprehensive assessment is conducted based on 5 factors: risk of bias, inconsistency, indirectness, imprecision, and publication bias. The quality of evidence is classified into 4 levels: high (⊕⊕⊕⊕), moderate (⊕⊕⊕◯), low (⊕⊕◯◯) , and very low (⊕◯◯◯). Sensitivity=0.90 (95% CI 0.85-0.94); specificity=0.92 (95% CI 0.87-0.95).

c CoE: certainty of evidence.

d Among the included studies, 2 had a high risk of bias regarding patient selection, 10 had an unclear risk of bias, and 5 had an unclear risk of bias regarding flow and timing.

e The 95% prediction intervals were significantly wider than the corresponding 95% CIs, and there was unexplained heterogeneity.

## Discussion

### Summary of Evidence

To the best of our knowledge, this study is the first to quantitatively synthesize the diagnostic performance of EEG-based ML models for SA detection via meta-analysis, while previous reviews only provided qualitative summaries [11]. Segment-level analyses demonstrated promising diagnostic performance, with a pooled sensitivity of 0.90 (95% CI 0.85‐0.94; *I*^2^=99.5%) and specificity of 0.92 (95% CI 0.87‐0.95; *I*^2^=99.8%), representing the average effect levels across all included studies, while the AUC was 0.95 (95% CI 0.92‐0.99). However, wide 95% prediction intervals for sensitivity (0.43‐0.99) and specificity (0.46‐0.99) suggest that the true effect may vary considerably across different research settings, and caution is warranted when extrapolating these findings to specific clinical contexts. Meta-regression analysis showed that EEG electrode configuration was significantly associated with both pooled sensitivity and specificity, whereas regional factors and validation strategies were associated with sensitivity and specificity, respectively. These findings suggest that differences in electrode configuration, regional background, and validation methods across studies may represent potential sources of heterogeneity. Subgroup analysis further showed that the multilead EEG model outperformed the single-lead model (sensitivity: 0.94 vs 0.87; specificity: 0.92 vs 0.90). However, the between-group differences did not reach statistical significance, and these findings should therefore be interpreted with caution. Studies conducted in Asian populations showed slightly better diagnostic performance than those in Western populations, and hold-out validation appeared to outperform cross-validation; however, these findings may be influenced by small subgroup sample sizes. However, the risk of bias in the included studies was low to moderate, and following a GRADE assessment, the overall certainty of the evidence was judged to be low, primarily due to high heterogeneity and risk of bias in some studies. Therefore, although the pooled estimates suggest promising diagnostic performance, confidence in the magnitude and generalizability of these estimates remains limited.

In exploring heterogeneity sources, the threshold effect was identified as a significant contributor, potentially attributable to differences in diagnostic task definitions and criteria [53]. Several studies did not stratify patients by disease severity or treat multiple condition categories as a single group [26]. In addition, most studies do not report specific thresholds or the rationale behind them, which limits further subgroup analysis. Subgroup analysis revealed that models using public datasets and automatic feature learning showed better performance than those using institution-specific datasets and manual feature extraction, respectively, though none of these differences reached statistical significance (*P*=.09 and *P*=.07, respectively), and the relevant conclusions should be interpreted with caution. Nevertheless, these trends suggest that the standardization of datasets and feature extraction strategies may influence model performance and the reproducibility of results. In addition, previous studies comparing portable monitoring systems with standard PSG have reported inconsistencies in signal synchronization and respiratory event recording [54]. This issue may stem from a lack of synchronization between the recording times of portable monitoring devices and PSG. This discrepancy can lead to inaccuracies in event annotation, which in turn can interfere with model training and result in biased performance evaluations [55]. In addition, sleep stage information has a significant impact on the performance of EEG-based SA detection models. The combined sleep stage and OSA screening model developed by Kang et al [56] found that in younger individuals, EEG features (K-complexes, spindles, and beta-band activity) differed significantly between rapid eye movement and nonrapid eye movement sleep, whereas in older adults, these differences were only observed during nonrapid eye movement sleep. This suggests that the interaction between sleep stages and EEG features varies by age, and models that fail to account for sleep stage information may exhibit inconsistent diagnostic performance across populations.

Multichannel EEG significantly outperformed single-channel EEG in both sensitivity and specificity. This aligns with the principle that multichannel configurations provide more comprehensive spatial information, capturing distributed brain network activity patterns associated with SA [57]. This finding aligns with the conclusions of Prucnal and Polak’s research [58]. Multichannel setups can partially offset noise interference or localized abnormal signals by integrating complementary signals from different functional brain regions [59]. Zhang et al [60] systematically investigated the performance differences between single-channel and multichannel data in automated sleep staging. Results indicated that as the complexity of classification tasks increased, the performance advantage of multichannel data became increasingly pronounced, further supporting the rationale for using multichannel EEG in complex sleep-related recognition tasks. However, constrained by factors such as channel configurations in public datasets, acquisition costs, and signal quality control, most studies favor single-channel EEG [61]. This tendency poses challenges for the practical application and widespread adoption of multichannel EEG. Consequently, channel selection strategies are crucial for balancing diagnostic performance with practical feasibility. Regarding channel selection, central channels—particularly C3-A2 and C4-A1—have been validated as effective choices due to their proximity to the sensorimotor cortex, where signals are closely associated with respiratory effort and microarousal [39,40,62]. Meanwhile, occipital-parietal pathways such as O1-A2 may more strongly reflect visual cortex activity and have also been reported as high-precision pathways [29,41]. Therefore, selectively incorporating occipital channels may provide supplementary information about sleep microstructure, particularly in distinguishing respiratory events associated with rapid eye movement.

PSG, as the gold standard for diagnosing SA, has reported sensitivity and specificity ranging from 80% to 97% and 85% to 97%, respectively [63]. However, its complex operation and requirement for specialized sleep laboratories limit widespread application [5]. Recent advances in ML offer a promising alternative solution. Gurrala et al [42] focused on EEG-based feature extraction and proposed a low-complexity method that outperformed earlier approaches using multimodal signals such as ECG, blood pressure, and respiratory data. These results support the feasibility of EEG-only ML approaches for SA detection. From a physiological perspective, EEG alterations triggered by respiratory events manifest as transient changes in spectral and temporal characteristics [64]. Segment-level analysis avoids information dilution caused by long-duration window averaging [65], supporting its use as a physiologically plausible approach.

Despite the favorable segment-level performance observed in this meta-analysis, translating segment-level prediction into clinically meaningful patient-level diagnosis remains a major challenge for EEG-based ML models. In clinical practice, SA severity is primarily determined using the apnea-hypopnea index (AHI), which is calculated based on the total number of apnea and hypopnea events per hour of sleep [66]. Therefore, accurate patient-level diagnosis requires not only reliable detection of respiratory events throughout the entire sleep recording but also accurate estimation of total sleep time and aggregation of detected events into clinically interpretable AHI values. However, many included studies focused primarily on segment-level classification performance and did not evaluate whether segment-level predictions could be reliably translated into patient-level AHI estimation. Furthermore, the included EEG-based studies primarily focus on apnea detection and do not clearly distinguish hypopnea events, which may further limit clinical applicability.

Although the overall performance of EEG-based ML detection models is satisfactory, pooled likelihood ratio maps suggest that they are insufficient for diagnosing or ruling out SA events on their own. Therefore, these models may be more suitable as adjunctive diagnostic or screening tools rather than standalone methods. Nevertheless, EEG-based AI systems demonstrate substantial potential for community-based and home-based sleep monitoring. Existing research indicates that continuous 5‐7 day community or home EEG monitoring can capture nocturnal variability in sleep patterns [67], avoid single-night PSG misdiagnosis, and enable early screening. Seol et al [68] validated a portable EEG device against PSG in 77 patients with OSA, reporting AUC values of 0.897 and 0.968 for EEG-based arousal index when screening for severe OSA (AHI ≥15 and ≥30, respectively). This indicates that consumer-grade EEG headbands can serve as effective preliminary screening tools in community settings, especially with automated analysis [69]. However, community health care facilities may face limitations due to equipment costs and technical personnel requirements [70]. Developing low-cost, portable simplified EEG devices combined with automated ML algorithms could overcome this bottleneck. Delimayanti et al [35] found that convolutional neural network models trained on raw EEG data outperformed those using fast Fourier transform–processed data, demonstrating the potential of convolutional neural networks for developing low-cost, accessible detection tools. Beyond improving diagnostic accessibility, AI-assisted EEG systems may contribute to integrated digital sleep health management pathways. Combining portable EEG devices with cloud-based AI analysis, remote telemonitoring, and electronic health record systems could establish proactive screening and longitudinal monitoring frameworks, facilitating early identification and timely referral of high-risk individuals [71]. Additionally, remote real-time EEG monitoring has been reported to reduce health care costs for brain disorders [72], though the cost-effectiveness of AI-assisted SA screening still requires further investigation.

Future research should prioritize establishing a standardized framework for model evaluation, threshold selection, and result reporting to improve consistency and comparability across studies. While leveraging the diagnostic advantages of multichannel EEG, future studies should also develop channel configuration strategies tailored to different clinical and real-world scenarios. In addition, AI-assisted EEG screening systems require further prospective validation in real-world settings, with emphasis on clinical utility, feasibility, and cost-effectiveness. Novel collaborative frameworks such as federated learning may facilitate multicenter data sharing and model training while preserving patient privacy, thereby improving model stability and generalizability across regions, populations, and health care systems[73]. In parallel, explainable AI methods (eg, Shapley Additive Explanations, Local Interpretable Model-Agnostic Explanations, and Gradient-Weighted Class Activation Mapping) may help address the “ black-box” limitation of DL models, improving interpretability and clinician trust in AI-assisted decision-making [74]. Finally, continued external validation, bias monitoring, and dynamic model updating remain essential to ensure fairness, stability, and long-term reliability in clinical practice [75].

### Limitations

This systematic review and meta-analysis has several limitations. First, during the data extraction phase, we recoded multiclass outcome measures into binary categories, which prevented further subgroup analyses based on diagnostic task types and limited the assessment of heterogeneity across different task scenarios. Second, quantitative synthesis could not be performed for some studies due to nonstandard reporting or missing key data. For studies with incomplete information, we reconstructed 2×2 contingency tables using statistical inference; this approach may have slightly affected the precision of the pooled effect sizes. We recommend that future studies present raw data completely and in a standardized manner to support higher-quality meta-analyses. Third, the majority of included studies used retrospective designs, and data sources were limited to public databases, which may introduce selection bias; consequently, study conclusions are difficult to generalize to broader clinical populations. Furthermore, and most critically, the vast majority of included studies did not undergo external validation. Most models were evaluated using only internal datasets or single-center data without independent external testing. This may lead to an overoptimistic assessment of the models’ diagnostic performance and significantly reduce their generalizability and practical value in real-world clinical settings. Therefore, future studies should prioritize rigorous external validation across multicenter settings and diverse populations to ensure the models’ stability and clinical translational value.

### Conclusions

Although EEG-based ML models demonstrate high diagnostic performance at the segment level, their ability to detect short apnea events does not necessarily reflect their performance in assessing overall disease severity (eg, AHI) during overnight monitoring. As a result, event-level metrics reported in current studies may overestimate their clinical screening and diagnostic value in real-world settings. Therefore, future research should prioritize overnight or patient-level external validation to better evaluate clinical applicability and generalizability. With advances in wearable EEG technology, EEG-based approaches remain promising as convenient and cost-effective tools for home sleep monitoring.

## Supplementary material

10.2196/93378 Multimedia Appendix 1 Details of the search strategy.

10.2196/93378 Multimedia Appendix 2 Full characteristics of the included studies.

10.2196/93378 Multimedia Appendix 3 Related materials.

10.2196/93378 Multimedia Appendix 4 ChatGPT.

10.2196/93378 Checklist 1 PRISMA-DTA checklist.

10.2196/93378 Checklist 2 PRISMA 2020 expanded checklist.

## Abbreviations

AHI: apnea-hypopnea index
AI: artificial intelligence
AUC: area under the curve
DL: deep learning
ECG: electrocardiogram
EEG: electroencephalogram
FN: false negative
FP: false positive
GRADE: Grading of Recommendations Assessment, Development and Evaluation
ML: machine learning
OSA: obstructive sleep apnea
PRISMA: Preferred Reporting Items for Systematic Reviews and Meta-Analyses
PRISMA-DTA: Preferred Reporting Items for Systematic Reviews and Meta-Analyses of Diagnostic Test Accuracy
PRISMA-S: Preferred Reporting Items for Systematic Reviews and Meta-Analyses Literature Search Extension
PROBAST+AI: Prediction Model Risk of Bias Assessment Tool for Artificial Intelligence
PSG: polysomnography
QUADAS-2: Quality Assessment of Diagnostic Accuracy Studies-2
SA: sleep apnea
TN: true negative
TP: true positive
