# Supplementary material for: Accuracy of Machine Learning Algorithms Based on Electroencephalogram in Sleep Apnea Detection: Systematic Review and Meta-Analysis
Source: J Med Internet Res. 2026 Jul 31;28:e93378. doi: 10.2196/93378 (PMC13427076; doi:10.2196/93378)
Supplement: Multimedia Appendix 1 [file jmir-v28-e93378-s001.doc]

| Pubmed | Search Details | Date | Results |
| --- | --- | --- | --- |
| #1 | (((((((((((((((((Artificial Intelligence) OR (Machine Intelligence)) OR (AI)) OR (Computer Reasoning)) OR (Computational Intelligence)) OR (Computer Vision Systems)) OR (Algorithms)) OR (machine learning)) OR (Deep Learning)) OR (artificial neural network)) OR (supervised machine learning)) OR (support vector machine)) OR (computer methodologies)) OR (information processing)) OR (computer‐aided)) OR (computer‐assisted)) OR (random forest)) OR (kNN) | 2026/4/22 | 3,262,351 |
| #2 | (((((((((Sleep Apnea Syndromes) OR (Sleep Apnea)) OR (Sleep Apneas)) OR (Sleep Hypopnea)) OR (Sleep Apnea, Mixed)) OR (Sleep Apnea, Obstructive)) OR (Mixed Central and Obstructive Sleep Apnea)) OR (Sleep-Disordered Breathing)) OR (OSAHS)) OR (Upper Airway Resistance Sleep Apnea Syndrome) | 2026/4/22 | 68,732 |
| #3 | (((EEG) OR (Electroencephalography)) OR (Electroencephalogram)) OR (Electroencephalograms) | 2026/4/22 | 226,405 |
| #4 | (((((((diagnostic accuracy) OR (diagnostic performance)) OR (diagnosis)) OR (detection)) OR (Sensitivity and Specificity)) OR (AUC)) OR (diagnostic performance)) OR (ROC) | 2026/4/22 | 14,419,589 |
| #1 AND #2 AND #3 AND #4 | ((((((((((((((((((((Artificial Intelligence) OR (Machine Intelligence)) OR (AI)) OR (Computer Reasoning)) OR (Computational Intelligence)) OR (Computer Vision Systems)) OR (Algorithms)) OR (machine learning)) OR (Deep Learning)) OR (artificial neural network)) OR (supervised machine learning)) OR (support vector machine)) OR (computer methodologies)) OR (information processing)) OR (computer‐aided)) OR (computer‐assisted)) OR (random forest)) OR (kNN)) AND ((((((((((Sleep Apnea Syndromes) OR (Sleep Apnea)) OR (Sleep Apneas)) OR (Sleep Hypopnea)) OR (Sleep Apnea, Mixed)) OR (Sleep Apnea, Obstructive)) OR (Mixed Central and Obstructive Sleep Apnea)) OR (Sleep-Disordered Breathing)) OR (OSAHS)) OR (Upper Airway Resistance Sleep Apnea Syndrome))) AND ((((EEG) OR (Electroencephalography)) OR (Electroencephalogram)) OR (Electroencephalograms))) AND ((((((((diagnostic accuracy) OR (diagnostic performance)) OR (diagnosis)) OR (detection)) OR (Sensitivity and Specificity)) OR (AUC)) OR (diagnostic performance)) OR (ROC)) | 2026/4/22 | 379 |

| Web of science | Search Details | Date | Results |
| --- | --- | --- | --- |
| #1 | Artificial Intelligence (Topic) OR Machine Intelligence (All Fields) OR AI (All Fields) OR Computer Reasoning (All Fields) OR Computational Intelligence (All Fields) OR Computer Vision Systems (All Fields) OR Algorithms (All Fields) OR machine learning (All Fields) OR Deep Learning (All Fields) OR artificial neural network (All Fields) OR supervised machine learning (All Fields) OR support vector machine (All Fields) OR computer methodologies (All Fields) OR information processing (All Fields) OR computer‐aided (All Fields) OR computer‐assisted (All Fields) OR random forest (All Fields) OR KNN (All Fields) | Tue Apr 22 2026 | 3,518,594 |
| #2 | Sleep Apnea Syndromes (Topic) OR Sleep Apnea (All Fields) OR Sleep Apneas (All Fields) OR Sleep Hypopnea (All Fields) OR Sleep Apnea, Mixed (All Fields) OR Sleep Apnea, Obstructive (All Fields) OR Mixed Central and Obstructive Sleep Apnea (All Fields) OR Sleep-Disordered Breathing (All Fields) OR OSAHS (All Fields) OR Upper Airway Resistance Sleep Apnea Syndrome (All Fields) | Tue Apr 22 2026 | 74,070 |
| #3 | Electroencephalography (Topic) OR Electroencephalogram (All Fields) OR Electroencephalograms (All Fields) OR EEG (All Fields) | Tue Apr 22 2026 | 123,384 |
| #4 | diagnostic accuracy (Topic) OR diagnostic performance (All Fields) OR diagnosis (All Fields) OR detection (All Fields) OR Sensitivity and Specificity (All Fields) OR ROC (All Fields) OR AUC (All Fields) | Tue Apr 22 2026 | 3,861,898 |
| #5 | #4 AND #3 AND #2 AND #1 | Tue Apr 22 2026 | 213 |

| Cochrane Libarary | Search details | Date | Results |
| --- | --- | --- | --- |
| #1 | （Sleep Apnea Syndromes):ti,ab,kw OR (Sleep Apnea):ti,ab,kw OR (Sleep Apneas):ti,ab,kw OR (Sleep Hypopnea):ti,ab,kw OR (Sleep Apnea, Mixed):ti,ab,kw | 2026/4/22 | 9,827 |
| #2 | (Sleep Apnea, Obstructive):ti,ab,kw OR (Mixed Central and Obstructive Sleep Apnea):ti,ab,kw OR (Sleep-Disordered Breathing):ti,ab,kw OR (OSAHS):ti,ab,kw OR (Upper Airway Resistance Sleep Apnea Syndrome):ti,ab,kw | 2026/4/22 | 8,636 |
| #3 | #1OR#2 | 2026/4/22 | 10,402 |
| #4 | (Artificial Intelligence):ti,ab,kw OR (Machine Intelligence):ti,ab,kw OR (Computer Reasoning):ti,ab,kw OR (AI):ti,ab,kw OR (Computational Intelligence):ti,ab,kw | 2026/4/22 | 15,691 |
| #5 | (Computer Vision Systems):ti,ab,kw OR (Algorithms):ti,ab,kw OR (machine learning):ti,ab,kw OR (Deep Learning):ti,ab,kw OR (artificial neural network):ti,ab,kw | 2026/4/22 | 24,674 |
| #6 | (supervised machine learning):ti,ab,kw OR (support vector machine):ti,ab,kw OR (computer methodologies):ti,ab,kw OR (information processing):ti,ab,kw OR (computer‐aided):ti,ab,kw | 2026/4/22 | 38,258 |
| #7 | (computer‐assisted):ti,ab,kw OR (random forest):ti,ab,kw OR (KNN):ti,ab,kw | 2026/4/22 | 23,924 |
| #8 | #4OR#5OR#6OR#7 | 2026/4/22 | 92,228 |
| #9 | (Electroencephalography):ti,ab,kw OR (Electroencephalogram):ti,ab,kw OR (Electroencephalogram):ti,ab,kw OR (EEG):ti,ab,kw | 2026/4/22 | 17,555 |
| #10 | (diagnostic accuracy):ti,ab,kw OR (diagnostic performance):ti,ab,kw OR (diagnosis):ti,ab,kw OR (Sensitivity and Specificity):ti,ab,kw | 2026/4/22 | 237,628 |
| #11 | (AUC):ti,ab,kw OR (ROC):ti,ab,kw | 2026/4/22 | 29,795 |
| #12 | #10OR#11 | 2026/4/22 | 260,748 |
| #13 | #3AND#8AND#9AND#12 | 2026/4/22 | 20 |

| Embase | Search details | Date | Results |
| --- | --- | --- | --- |
| #1 | sleep apnea syndromes'/exp OR 'sleep apnea syndromes' OR 'sleep apnea':ab,ti OR 'sleep apneas':ab,ti OR 'sleep apnea, mixed':ab,ti OR 'sleep apnea, obstructive':ab,ti OR ('mixed central':ab,ti AND 'obstructive sleep apnea':ab,ti) OR 'sleep-disordered breathing':ab,ti OR osahs:ab,ti OR 'upper airway resistance sleep apnea syndrome':ab,ti | 2026/4/22 | 129,978 |
| #2 | artificial intelligence'/exp OR 'artificial intelligence' OR (artificial AND ('intelligence'/exp OR intelligence)) OR 'machine intelligence':ab,ti OR ai:ab,ti OR 'computer reasoning':ab,ti OR 'computational intelligence':ab,ti OR algorithms:ab,ti OR 'computer vision systems':ab,ti OR 'deep learning':ab,ti OR 'machine learning':ab,ti OR 'artificial neural network':ab,ti OR 'supervised machine learning':ab,ti OR 'computer methodologies':ab,ti OR 'support vector machine':ab,ti OR 'information processing':ab,ti OR computer‐aided:ab,ti OR computer‐assisted:ab,ti OR 'random forest':ab,ti OR knn:ab,ti | 2026/4/22 | 786,375 |
| #3 | electroencephalography'/exp OR 'electroencephalography' OR electroencephalogram:ab,ti OR electroencephalograms:ab,ti OR eeg:ab,ti | 2026/4/22 | 307,524 |
| #4 | diagnosis'/exp OR diagnosis OR 'diagnostic accuracy':ab,ti OR detection:ab,ti OR (sensitivity:ab,ti AND specificity:ab,ti) OR 'diagnostic performance':ab,ti OR auc:ab,ti OR roc:ab,ti | 2026/4/22 | 13,337,805 |
| #5 | #4 AND #3 AND #2 AND #1 | 2026/4/22 | 397 |

| IEEE | Search details | Date | Results |
| --- | --- | --- | --- |
| #1 | ("All Metadata":Sleep Apnea Syndromes) OR ("All Metadata":Sleep Apnea) OR ("All Metadata":Sleep Apneas) OR ("All Metadata":Sleep Hypopnea) OR ("All Metadata":Sleep Apnea, Mixed) OR ("All Metadata":Sleep Apnea, Obstructive) OR ("All Metadata":Mixed Central and Obstructive Sleep Apnea) OR ("All Metadata":Sleep-Disordered Breathing) OR ("All Metadata":OSAHS) OR ("All Metadata":Upper Airway Resistance Sleep Apnea Syndrome) | 2026/4/22 | 3,585 |
| #2 | ("All Metadata":Electroencephalography) OR ("All Metadata":Electroencephalogram) OR ("All Metadata":Electroencephalograms) OR ("All Metadata":EEG) | 2026/4/22 | 36,626 |
| #3 | ("All Metadata":Artificial Intelligence) OR ("All Metadata":Machine Intelligence) OR ("All Metadata":machine learning) OR ("All Metadata":Deep Learning) OR ("All Metadata":artificial neural network) OR ("All Metadata":supervised machine learning) AND ("All Metadata":support vector machine) OR ("All Metadata":computer¿aided) OR ("All Metadata":computer¿assisted) OR ("All Metadata":random forest) OR ("All Metadata":KNN) | 2026/4/22 | 1,030,836 |
| #4 | ("All Metadata":diagnostic accuracy) OR ("All Metadata":diagnostic performance) OR ("All Metadata":diagnosis) OR ("All Metadata":detection) OR ("All Metadata":Sensitivity and Specificity) OR ("All Metadata":AUC) OR ("All Metadata":ROC) | 2026/4/22 | 986,180 |
| #5 | ((((((All Metadata:Artificial Intelligence) OR (All Metadata:Machine Intelligence) OR (All Metadata:machine learning) OR (All Metadata:Deep Learning) OR (All Metadata:artificial neural network) OR (All Metadata:supervised machine learning) AND (All Metadata:support vector machine) OR (All Metadata:computer¿aided) OR (All Metadata:computer-assisted) OR (All Metadata:random forest) OR (All Metadata:KNN))) AND ((All Metadata:diagnostic accuracy) OR (All Metadata:diagnostic performance) OR (All Metadata:diagnosis) OR (All Metadata:detection) OR (All Metadata:Sensitivity and Specificity) OR (All Metadata:AUC) OR (All Metadata:ROC))) AND ((No Keywords Specified))) AND ((All Metadata:Sleep Apnea Syndromes) OR (All Metadata:Sleep Apnea) OR (All Metadata:Sleep Apneas) OR (All Metadata:Sleep Hypopnea) OR (All Metadata:Sleep Apnea, Mixed) OR (All Metadata:Sleep Apnea, Obstructive) OR (All Metadata:Mixed Central and Obstructive Sleep Apnea) OR (All Metadata:Sleep-Disordered Breathing) OR (All Metadata:OSAHS) OR (All Metadata:Upper Airway Resistance Sleep Apnea Syndrome))) AND ((All Metadata:Electroencephalography) OR (All Metadata:Electroencephalogram) OR (All Metadata:Electroencephalograms) OR (All Metadata:EEG)) | 2026/4/22 | 265 |

Scopus 520

( TITLE-ABS-KEY ( "Sleep Apnea Syndromes" OR "Sleep Apnea" OR "Sleep Apneas" OR "Sleep Hypopnea" OR "Sleep Apnea, Mixed" OR "Sleep Apnea, Obstructive" OR "Mixed Central and Obstructive Sleep Apnea" OR "Sleep-Disordered Breathing" OR "OSAHS" OR "Upper Airway Resistance Sleep Apnea Syndrome" ) AND TITLE-ABS-KEY ( "EEG" OR "Electroencephalography" OR "Electroencephalogram" OR "Electroencephalograms" ) AND TITLE-ABS-KEY ( "diagnostic accuracy" OR "diagnostic performance" OR "diagnosis" OR "detection" OR "Sensitivity and Specificity" OR "AUC" OR "ROC" ) AND TITLE-ABS-KEY ( "Artificial Intelligence" OR "Machine Intelligence" OR "AI" OR "Computer Reasoning" OR "Computational Intelligence" OR "Computer Vision Systems" OR "Algorithms" OR "machine learning" OR "Deep Learning" OR "artificial neural network" OR "supervised machine learning" OR "support vector machine" OR "computer methodologies" OR "information processing" OR "computer‐aided" OR "computer‐assisted" OR "random forest" OR "kNN" ) ）

Clinicaltrails 7

"Sleep Apnea Syndromes" OR "Sleep Apnea" OR "Sleep Apneas" OR "Sleep Hypopnea" OR "Sleep Apnea, Mixed" OR "Sleep Apnea, Obstructive" OR "Mixed Central and Obstructive Sleep Apnea" OR "Sleep-Disordered Breathing" OR "OSAHS" OR "Upper Airway Resistance Sleep Apnea Syndrome" | Other terms: (Artificial Intelligence) OR (Machine Intelligence) OR (AI) OR (Computer Reasoning) OR (Computational Intelligence) OR (Computer Vision Systems) OR (Algorithms) OR (machine learning) OR (Deep Learning) OR (artificial neural network) OR (supervised machine learning) OR (support vector machine) OR (computer methodologies) OR (information processing) OR (computer-aided) OR (computer-assisted) OR (random forest) OR (kNN) | (EEG) OR (Electroencephalography) OR (Electroencephalogram) OR (Electroencephalograms)

| EBSCO | Search details | Date | Results |
| --- | --- | --- | --- |
| #1 | Sleep Apnea Syndromes OR Sleep Apnea OR Sleep Apneas OR Sleep Hypopnea OR Sleep Apnea, Mixed OR Sleep Apnea, Obstructive OR (Mixed Central and Obstructive Sleep Apnea) OR Sleep-Disordered Breathing OR OSAHS OR Upper Airway Resistance Sleep Apnea Syndrome | 2026/4/22 | 23,274 |
| #2 | Artificial Intelligence OR Machine Intelligence OR Sleep Apneas OR AI OR Computer Reasoning OR Computer Vision Systems OR Algorithms OR machine learning OR Deep Learning OR artificial neural network OR supervised machine learning OR support vector machine | 2026/4/22 | 1,186,254 |
| #3 | computer methodologies OR information processing OR computer‐aided OR computer‐assisted OR random forest OR KNN | 2026/4/22 | 597,294 |
| #4 | #2 OR #3 | 2026/4/22 | 1,659,760 |
| #5 | EEG OR Electroencephalography OR Electroencephalogram OR Electroencephalograms | 2026/4/22 | 54,050 |
| #6 | diagnostic accuracy OR diagnostic performance OR diagnosis OR detection OR (Sensitivity and Specificity) OR AUC OR ROC | 2026/4/22 | 2,285,150 |
| #7 | #1 AND #4 AND #5 AND #6 | 2026/4/22 | 113 |
