# Supplementary material for: Accuracy of Machine Learning Algorithms Based on Electroencephalogram in Sleep Apnea Detection: Systematic Review and Meta-Analysis
Source: J Med Internet Res. 2026 Jul 31;28:e93378. doi: 10.2196/93378 (PMC13427076; doi:10.2196/93378)
Supplement: Multimedia Appendix 2 [file jmir-v28-e93378-s002.docx]

| Author | Year | Article name | Text type | Journal name | Country | Research type | Purpose of research | Database | Detection Level | sample size | patient | healthy individuals | training set | validation set | test set | internal and external validation | gender | age | Diagnostic gold standard | EEG channel | Channel name | duration | Signal fragment length | Sample size per time slot (subframe length) | frequency（HZ） | feature source | Training/Validation/Testing（%） | validation method | Preprocessing | Feature extraction | Feature selection | Algorithm type | Algorithm name |  |
| --- | --- | --- | --- | --- | --- | --- | --- | --- | --- | --- | --- | --- | --- | --- | --- | --- | --- | --- | --- | --- | --- | --- | --- | --- | --- | --- | --- | --- | --- | --- | --- | --- | --- | --- |
| Cheng et al [26] | 2023 | Multiple-instance learning for EEG based OSA event detection | Journal article | Biomedical Signal Processing and Control | China | Retrospective study | detect OSA | UCDDB | epoch | 25（Individual） | 2680（Frame-level） | 7197 | / | / | / | internal and external validation | 21M/4F | 36-58 | PSG | Single-channel | C3-A2 | all night | 60s | / | 128 | time domain, frequency domain | / | 5-fold cross-validation | / | Automatic Extraction | MIL mapping function (based on self-attention mechanism) | DL | EEG-MIL |  |
|  |  |  |  |  |  |  |  | ISRUC |  | 61（Individual） | 4099 | 19629 |  |  |  |  | 32M/29F |  |  |  |  |  |  |  |  |  |  |  |  |  |  |  |  |  |
|  |  |  |  |  |  |  |  | Local hospital |  | 35（Individual） | 4211 | 8960 |  |  |  |  | 27M/8F |  |  |  |  |  |  |  |  |  |  |  |  |  |  |  |  |  |
| Jiang et al [29] | 2018 | A Multi-Scale Parallel Convolutional Neural Network for Automatic Sleep Apnea Detection Using Single-Channel EEG Signals | Conference Papers | 2018 11th International Congress on Image and Signal Processing, BioMedical Engineering and Informatics (CISP-BMEI) | China | Retrospective study | detect SA | MIT-BIH | epoch | 16（Individual）2650（Event Segment） | 1615 | 1035 | / | / | / | internal | M | 32-56 | PSG | Single-channel | O2-A1、C4-A1、C3-O1 | all night | 30s | 7500 | 250 | Time-frequency domain | 80/20 | 5-fold cross-validation | Finite Impulse Response (FIR) Bandpass Filter for Noise Reduction | Time-frequency image generated by Short-time Fourier Transform | Model automatically learns multi-scale features | DL | MSPCNN |  |
| Emin Tagluk and Sezgin [30] | 2011 | A new approach for estimation of obstructive sleep apnea syndrome | Journal article | Expert Systems with Applications | Turkey | Retrospective study | detect OSA | / | epoch | 20（Individual） | 15（Individual）2500 | 5（individual）2500 | 300(150 SA,150 normal) | Select 20 samples from the training set (15 SA, 15 normal) | 2350SA，2350normal | internal | / | 36±8 | PSG | Single-channel | C3-A2 | all night(7-8h） | 10s | 2560 | 256 | Frequency Domain (Dual Spectrum Analysis) | / | Fixed Sample Validation Method | Han Ning window (width 0.1s) processing; preprocessing before dual-spectrum analysis | QPC characteristics | Dual-spectrum analysis, QPC quantification | ML | ANN |  |
| Lin et al [31] | 2006 | A NEW APPROACH FOR IDENTIFYING SLEEP APNEA SYNDROME USING WAVELET TRANSFORM AND NEURAL NETWORKS | Journal article | Biomedical Engineering: Applications, Basis and Communications | China | Retrospective study | detect SA | MIT-BIH | epoch | / | / | / | / | / | / | internal | / | / | PSG | Single-channel | C3-O1 | 4h | 30s | / | 250 | Frequency domain (wavelet coefficients) | / | Hold-out validation | / | DWT | / | ML | ANN |  |
| Zhang et al [32] | 2025 | An EEG Screening Method for Severe Obstructive Sleep Apnea Based on Limited Penetrable Difference Visibility Graph and Graph Convolutional Network | Journal article | IEEE Journal of Biomedical and Health Informatics | China | Retrospective study | Screening for severe OSA | The Seventh Affiliated Hospital of Sun Yat-sen University | individuality | 88（Individual） | 37 moderate, 26 severe | 25 | / | / | / | internal | / | 31-61 | PSG | Multi-channel | F3、F4、C3、C4、O1、O2 | 6-hour sleep segment | 5s(relative power calculation window), 30s(SWA calculation window) | / | 200 | Network Characteristics | / | 10-fold nested cross-validation | Bandpass filter (FIR filter) | LPDVG_wSEN，A separate traditional slow wave activity (SWA) was established as a contrast feature. | parameter optimization | DL | GCN |  |
| Wang et al [33] | 2021 | An Efficient Method to Detect Sleep Hypopnea- Apnea Events Based on EEG Signals | Journal article | IEEE Access | China | Retrospective study | detect SA | Tianjin Chest Hospital | epoch | 30（Individual） | 19 severe (15 males/4 females), 7 moderate, 4 mild; 407+405 (event segments) | 812（Event Segment） | 1218 | 406 | / | internal | 23M/7F | 34-78 | PSG | Multi-channel | C3-A2 and C4-A1 | all night | 10s | / | 100 | Time-frequency domain | 75/25 | Hold-out validation | The Bartworth filter is decomposed into five bands for discrete wavelet transform | Entropy | SVM-RFE feature selection | ML | random forest |  |
| Prucnal and Polak[34] | 2018 | Analysis of Features Extracted from EEG Epochs by Discrete Wavelet Decomposition and Hilbert Transform for Sleep Apnea Detection | Conference Papers | 2018 40th Annual International Conference of the IEEE Engineering in Medicine and Biology Society (EMBC) | Poland | Retrospective study | Three-category classification: NB/OAH/CAH | UCDDB | epoch | 1320(Event Segment） | / | / | 924 | 198 | 198 | internal | / | / | PSG | Single-channel | C3-A2 | / | 30s | / | 128 | Time-frequency domain | 70/15/15 | / | Using db3 (Daubechies 3-order) wavelets, the EEG signals of each epoch were decomposed into four levels, resulting in five frequency sub-bands. | mean, standard deviation, skewness, kurtosis, median | Analysis of Variance (ANOVA) | DL | FFNN |  |
|  |  |  |  |  |  |  |  |  |  |  |  |  |  |  |  |  |  |  |  |  |  |  |  |  |  |  |  |  |  |  |  |  |  |  |
| Delimayanti et al [35] | 2025 | Automated Sleep Apnea Detection Using CNNs: Insights into the Impact of FFT Feature Extraction on EEG Signals | Journal article | Journal of Advances in Information Technology | India | Retrospective study | detect SA | CAP Sleep database | epoch | 5（Individual）60（Event Segment） | 2（Individual）30（Event Segment） | 3（individual）30 | 42 | 6 | 12 (6 normal, 6 SA) | internal | / | / | PSG | Multi-channel | Fp1-F3, F3-C3, C3-P3, P3-O1 and/or Fp2-F4, F4-C4, C4-P4, P4-O2（遵循 10-20 国际系统） | all night(7-8h） | 30min |  | 256→64 | Time domain, frequency domain | 70/10/20 | / | data standardization | 1. Model 1 (raw EEG): 1D convolutional layer automatically extracts temporal features; 2. Model 2 (FFT processing): Fast Fourier Transform extracts frequency-domain features | Model automatically learns key features | DL | CNN |  |
| Zhou et al [36] | 2015 | Automatic detection of sleep apnea based on EEG detrended fluctuation analysis and support vector machine | Journal article | Journal of Clinical Monitoring and Computing | China | Retrospective study | detect SA | MIT-BIH | epoch | 12（Individual）720（Event Segment） | 6（Individual）360（Event Segment） | 6（individual）360 | / | / | / | internal | Male in the patient group, mixed in the healthy group | 32-51 | PSG | Single-channel | C3-O1 | / | 30s | / | / | Nonlinear characteristics | / | Ten independent trials | Threshold denoising wavelet using db4 function for signal denoising | The trend fluctuation analysis (DFA2) was used to extract the scale index α, which reflects the long-range power-law correlation of EEG signals. | DFA scaling exponent | ML | SVM |  |
| Saha et al [37] | 2019 | Automatic detection of sleep apnea events based on inter‐band energy ratio obtained from multi‐band EEG signal | Journal article | Healthcare Technology Letters | Bangladesh | Retrospective study | detect SA | UCDDB | frame | 5（Individual）1706（Event Segment） | 853 | 853 | / | / | / | internal | / | Average 50.8 | PSG | Multi-channel | C3–A2、C4–A1 | / | 10s | / | 128 | frequency domain | / | Leave-one-out cross-validation | Spectral filtering in the fast Fourier transform domain divides the EEG signal into five frequency bands, removes DC offset (frame mean subtraction), performs intra-frame normalization (relative to the maximum value of the frame), and applies time-domain averaging of dual-channel data (optional for noise reduction). | interband energy ratio | / | ML | KNN |  |
| Gupta et al [38] | 2020 | Automatic Detection of Sleep Apnea Using Sub-Band Features from EEG Signals | Conference Papers | 2020 3rd International Conference on Signal Processing and Information Security (ICSPIS) | India | Retrospective study | detect SA | UCDDB | frame | 5（Individual）1706（Event Segment） | 853 | 853 | / | / | / | internal | / | / | PSG | Multi-channel | C3–A2、C4–A1 | / | 10s |  | 128 | Time domain, frequency domain | 80/20 | 5-fold cross-validation | The 20th-order infinite impulse response Butterworth bandpass filter divides the EEG signal into five frequency bands. | Energy, entropy, kurtosis, and mean absolute deviation | Feature Ranking | ML | Ensemble (Bagged Trees) |  |
| Wang et al [39] | 2022 | BI - Directional long short-term memory for automatic detection of sleep apnea events based on single channel EEG signal | Journal article | Computers in Biology and Medicine | China | Retrospective study | detect SA | Tianjin Chest Hospital | epoch | 13896（Event Segment） | 7573 | 6323 |  | 1390 | / | internal | / | 37–78 | PSG | Single-channel | C3-A2 | all night | 10s | / | 100 | Model autonomous learning of temporal features | 90 10 | 10-fold cross-validation | A 4th-order IIR filter was applied to the EEG signal in the 4–45 Hz range, followed by band-pass filtering. The EEG signals from the same channel were then normalized to the (0,1) range. | Automatic learning of temporal features of EEG signals by BI-LSTM model | model self-filtering | DL | BI-LSTM |  |
|  |  |  |  |  |  |  |  |  |  | 13896（Event Segment） | 7573 | 6323 |  | 1390 | / |  | / | 37–78 |  |  | C4-A1 |  |  |  |  |  |  |  |  |  |  |  |  |  |
| Zhao et al [40] | 2021 | Classification of sleep apnea based on EEG sub-band signal characteristics | Journal article | Scientific Reports | China | Retrospective study | Three-category classification: NB/OAH/CAH | Tianjin Chest Hospital | epoch | 30（Individual） | OSA 1229/CSA 812（Event Segment） | 1418 | / | / | / | internal | 23M/7F | 37–78 | PSG | Multi-channel | C3-A2 and C4-A1 | / | / | / | 100 | Statistical Characteristics | / | 10-fold cross-validation | The infinite impulse response Butterworth bandpass filter decomposes the EEG signal into five sub-bands | entropy variance | neighbor component analysis (NCA) | ML | RF |  |
|  |  |  |  |  |  |  |  |  |  |  |  |  |  |  |  |  |  |  |  |  |  |  |  |  |  |  |  |  |  |  |  |  |  |  |
| Bonner et al [41] | 2024 | Deep Learning-based EEG Analysis for Sleep Apnea Detection | Conference Papers | 2024 Systems and Information Engineering Design Symposium (SIEDS) | United States | Retrospective study | detect SA | MIT-BIH | epoch | 15（Individual） | / | / | / | / | / | internal | M | 32-56 | PSG | Single-channel | C3-01、C4-A1、O2-A1 | / | 30s | / | / | Time domain | / | / | / | / | end to end automation | DL | TCNN |  |
| Gurrala et al [42] | 2021 | Detection of Sleep Apnea Based on the Analysis of Sleep Stages Data Using Single Channel EEG | Journal article | Traitement du Signal | India | Retrospective study | detect SA | MIT-BIH | epoch | 18（Individual）10216（Event Segment） | / | / | / | / | / | internal | / | / | PSG | Single-channel | / | 6h | 30s | 7500 | 250 | Time domain, frequency domain | / | / | Adaptive noise-complete set empirical mode decomposition is employed for artifact removal, while wavelet transform (WT) is utilized for signal filtering. | Sleep Stage (SS), Sleep Stage Occurrence Frequency (OSS), Relative Power of Period (RPE) | / | ML | Ensemble Bagged Tree |  |
| Taran et al [43] | 2021 | Detection of sleep apnea events using electroencephalogram signals | Journal article | Applied Acoustics | India | Retrospective study | detect SA | MIT-BIH | epoch | 2142（Event Segment） | 947 | 1195 | / | / | / | internal | / | Average 43 | PSG | Single-channel | / | / | / | / | 256 | Complexity Features | / | 10-fold cross-validation | The EEG signal is decomposed into 16 subbands using a 15-level TQWT. | LZC (non-parametric complexity metric), 1D convolutional layer automatically extracts temporal features | Model automatically learns key features | ML | subspace-KNN |  |
| Barnes et al [27] | 2022 | Detection of sleep apnea from single-channel electroencephalogram (EEG) using an explainable convolutional neural network (CNN) | Journal article | PLOS ONE | New Zealand | Retrospective study | detect SA | SHHS | epoch | 2650（Individual） | / | / | / | / | / | internal and external validation | / | / | PSG | Single-channel | C4-A1 | all night | 30s | / | 125/128 | Single-channel EEG signal | 10-09-1981 | 10-fold cross-validation | Low-pass filtering, downsampling, z-score normalization, segmentation, label annotation, majority class undersampling (training set and validation set) | network automatic learning | / | DL | CNN |  |
|  |  |  |  |  |  |  |  | UCDDB |  | 25（Individual） |  |  |  |  |  |  |  |  | PSG |  |  |  |  |  | 128 |  |  |  |  |  |  |  |  |  |
|  |  |  |  |  |  |  |  | MIT-BIH |  | 16（Individual） |  |  |  |  |  |  |  |  | PSG |  |  |  |  |  | 250 |  |  |  |  |  |  |  |  |  |
| Khan et al [44] | 2023 | ESAD: Expert System for Apnea Detection Using Enhanced DWT Feature Extraction and Machine Learning Algorithms | Conference Papers | 2023 14th International Conference on Computing Communication and Networking Technologies (ICCCNT) | India | Retrospective study | detect SA | SHHS 1 | individuality | 4881（ Individual） | 2434 | 2447 | / | / | / | internal | / | 40 and above | PSG | Single-channel | C4-A1 | / | / | / | / | Time domain, frequency domain | / | / | The discrete wavelet transform (DWT) of an 8-order Daubechies wavelet is used to preprocess and decompose EEG data, dividing it into five frequency sub-bands. | Minimum, Maximum, Average, Variance, Kurtosis, Energy, Power, Standard Deviation, and Root Mean Square | The isolated forest algorithm (IF) detects and removes outliers | ML | SVM（With RBF） |  |
| Mahmud et al [28] | 2019 | Real Time Sleep Apnea Event Detection with Deep Neural Network | Conference Papers | 2019 IEEE International Conference on Biomedical Engineering, Computer and Information Technology for Health (BECITHCON) | Bangladesh | Retrospective study | detect SA | UCDDB | epoch | 12（Individual） | / | / | 7 | 5 | / | external | / | / | PSG | Multi-channel | C3-A2 and C4-Al | all night | 1s | / | 128 | Time domain | / | Independent Dataset Validation | Segment non-overlapping frames in 1 second; discard some non-apnea frames to balance category proportions | 1D convolutional layer automatically extracts deep features | Model Self-Learning | ML | CNN |  |
| Prucnal and Polak [45] | 2023 | Single-channel EEG processing for sleep apnea detection and differentiation | Journal article | Metrology and Measurement Systems | Poland | Retrospective study | Three-category classification: NB/OAH/CAH | UCDDB | epoch | 25（Individual）4119（Event Segment） | / | / | / | / | / | internal | 21M/4F | / | PSG | Single-channel | C3-A2 | / | 30s | / | 128 | Time-frequency domain | / | 32-fold cross-validation | A 17th-order Chebyshev-type zero-phase low-pass filter (cutoff frequency 45 Hz) was employed, followed by a 2nd-order zero-phase notch filter. | Seven methods, including HHT (351 initial features) and DWT+HT (135 initial features), were ultimately screened to identify 387 features. | ReliefF algorithm | DL | SVM |  |
|  |  |  |  |  |  |  |  |  |  |  |  |  |  |  |  |  |  |  |  |  |  |  |  |  |  |  |  |  |  |  |  |  |  |  |
|  |  |  |  |  |  |  |  |  |  |  |  |  |  |  |  |  |  |  |  |  |  |  |  |  |  |  |  |  |  |  |  |  |  |  |
|  |  |  |  |  |  |  |  |  |  |  |  |  |  |  |  |  |  |  |  |  |  |  |  |  |  |  |  |  |  |  |  |  |  |  |
|  |  |  |  |  |  |  |  |  |  |  |  |  |  |  |  |  |  |  |  |  |  |  |  |  |  |  |  |  |  |  |  |  |  |  |
|  |  |  |  |  |  |  |  |  |  |  |  |  |  |  |  |  |  |  |  |  |  |  |  |  |  |  |  |  |  |  |  |  |  |  |
| Wijaya et al [46] | 2024 | Sleep Apnea Identification Based on EEG Signals Using Hybrid Spatio-Temporal Deep Learning | Conference Papers | 2024 International Conference on Computer, Control, Informatics and its Applications (IC3INA) | Indonesia | Retrospective study | Three-category classification: NB/OAH/CAH | MGH 2018 | epoch | 25845（Event Segment） | / | / | 20676 | 5169 | / | internal | / | / | PSG | Multi-channel | C3-M2 and C4-M1 | all night（6-8h） | 30s | / | 200 | Spatio-temporal characteristics | 80/20 | / | Extract the effective frequency band of 0.5-30Hz using the Daubechies db4 wavelet transform | The 1D-CNN (1×3 filter, stride 1) extracts spatial features, while the maximum pooling (1×2, stride 3) reduces dimensionality; the model achieves end-to-end self-learning. | CNN and GRU Collaboratively Screen Valid Features | DL | CNN-GRU |  |
|  |  |  |  |  |  |  |  |  |  |  |  |  |  |  |  |  |  |  |  |  |  |  |  |  |  |  |  |  |  |  |  |  |  |  |
| Bhalerao and Pachori [47] | 2022 | Sparse spectrum based swarm decomposition for robust nonstationary signal analysis with application to sleep apnea detection from EEG | Journal article | Biomedical Signal Processing and Control | India | Retrospective study | detect SA | MIT-BIH | epoch | 14（Individual） | 4020epoch | 3978 | / | / | / | internal | M | Average 43 | PSG | Multi-channel | C3/O1、C4/A1 and O2/A1 | all night | 10s | / | 250→128 | Time domain, frequency domain, time-frequency domain | / | Leave-one-out cross-validation | A tenth-order Butterworth bandpass filter (passband frequency of 2 Hz to 45 Hz) is employed to eliminate various artifacts. Additionally, a 60 Hz notch filter is utilized to remove power line interference in SSDM. | time domain, fractal dimension, frequency domain, time-frequency domain | Screening significant features by t-test (p<0.05), selecting 26 dimensions from 72 | DL | CNN |  |
|  |  |  |  |  |  |  |  |  |  |  |  |  |  |  |  |  |  |  |  |  |  |  |  |  |  |  |  |  |  |  |  |  |  |  |
| Shahnaz et al [19] | 2016 | Sub-frame based apnea detection exploiting delta band power ratio extracted from EEG signals | Conference Papers | TENCON 2016 - 2016 IEEE Region 10 Conference | Bangladesh | Retrospective study | detect SA | MIT-BIH | frame | 14（Individual）2720（Event Segment） | 1360 | 1360 | / | / | / | internal | / | / | PSG | / | / | / | 30s(main frame), 10s(subframe, with 1 second overlap) | / | / | frequency domain | / | 2-fold cross-validation | DB4 is selected as the wavelet type for denoising and smoothing. The threshold selection rule adopts heuristic. | Mean Standard Deviation | δ-band Power Ratio (DBPR) | ML | SVM |  |
|  |  |  |  |  |  |  |  |  |  |  |  |  |  |  |  |  |  |  |  |  |  |  |  |  |  |  |  |  |  |  |  |  |  |  |
| Taran et al [48] | 2017 | TEO separated AM-FM components for identification of apnea EEG signals | Conference Papers | 2017 IEEE 2nd International Conference on Signal and Image Processing (ICSIP) | India | Retrospective study | detect SA | MIT-BIH | epoch | 16（Individual）2124（Event Segment） | 947 | 1195 | / | / | / | internal | / | / | PSG | Single-channel | / | / | 30s | / | 256 | Time-frequency domain | / | / | network automatic learning | Separate the AM (Amplitude Modulation) and FM (Frequency Modulation) components of each IMF using the Teager Energy Operator (TEO). | IMF conspicuousness | ML | LS-SVM(RBF) IMF1 |  |
| Sharifi and Fakharzadeh [49] | 2025 | Algorithm for EEG—Based Sleep—Wake Classification Toward Sleep Apnea Detection | Conference Papers | 2025 32nd National and 10th International Iranian Conference on Biomedical Engineering (ICBME) | Iran | Retrospective study | detect SA | MIT-BIH | epoch | 3640 | 991 | 2649 |  |  |  | internal | M |  | PSG | Single-channel | C4-A1 |  | 30s |  | 250 | Time-frequency domain | 80/20 | 10-fold cross-validation | Noise filtering, signal segmentation | Entropy (manual), mean, standard deviation | / | ML | RF |  |
| Saha et al [50] | 2026 | An Apnea Detection Method Based on Temporal Feature Variational Pattern of Multi-band EEG Signal Incorporating Sleep Stage Information | Journal article | Circuits, Systems, and Signal Processing | Bangladesh | Retrospective study | detect SA | MIT-BIH | epoch | 16（Individual）3000（Event Segmen） | 1500 | 1500 |  |  |  | internal | M | 32-56 | PSG | Single-channel | C3-A2 |  | 30s |  | 250 | Time domain |  | 5-fold cross-validation | DC Offset Removal, Amplitude Normalization | Mean, standard deviation, median | LPPA、LVA、LMAD | ML | KNN |  |
|  |  |  |  |  |  |  |  | UCDDB | epoch | 25（Individual）4700（Event Segmen | 2350 | 2350 |  |  |  |  | 21M/4F | 28-68 | PSG | Single-channel | O2-A1, C4-A1, and C3-O1. |  | 15s |  | 128 | Time domain |  | 5-fold cross-validation |  |  |  |  |  |  |
| Band and Deshmukh [51] | 2026 | Heuristic Deep Learning Framework for EEG-based Sleep Apnea Event Classification | Journal article | International Research Journal of Multidisciplinary Scope | India | Retrospective study | detect SA | Sleep EDF Dataset | epoch | 400 | / | / |  |  |  | internal | / | / | PSG | Single-channel | (Fpz-Cz) |  | 30s |  | 100 | Time-frequency domain | 70/30 | 10-fold cross-validation | Using the discrete wavelet transform (DWT) with db4 (Dabush 4th-order) mother wavelets, each segment of the EEG signal is decomposed into five sub-bands to extract specific frequency information of research value, thereby completing the band-splitting and normalization of the EEG signals. | Mean, standard deviation, skewness, variance, and kurtosis; frequency-domain features include band power, relative power, and spectral entropy; nonlinear features include approximate entropy, sample entropy, and the Higuchi fractal dimension | Heuristic Domain-Driven Feature Selection | DL | 1D-CNN |  |

### References

19. Shahnaz C, Minhaz AT, Ahamed S. Sub-frame based apnea detection exploiting delta band power ratio extracted from EEG signals. In: Shahnaz C, Minhaz AT, Ahamed ST, editors. Presented at: TENCON 2016—2016 IEEE Region 10 Conference; Nov 22-25, 2016; Singapore. 2016.[doi: 10.1109/TENCON.2016.7847987]

26. Cheng L, Luo S, Li B, Liu R, Zhang Y, Zhang H. Multiple-instance learning for EEG based OSA event detection. Biomed Signal Process Control. Feb 2023;80:104358. [doi: 10.1016/j.bspc.2022.104358]

27. Barnes LD, Lee K, Kempa-Liehr AW, Hallum LE. Detection of sleep apnea from single-channel electroencephalogram (EEG) using an explainable convolutional neural network (CNN). PLoS One. 2022;17(9):e0272167. [doi: 10.1371/ journal.pone.0272167] [Medline: 36099242]

28. Mahmud T, Aeioub Ansary M, Mahmud TI, Khan IA, Fattah SA. Real time sleep apnea event detection with deep neural network. Presented at: 2019 IEEE International Conference on Biomedical Engineering, Computer and Information Technology for Health (BECITHCON); Nov 28-30, 2019; Dhaka, Bangladesh. 2019.[doi: 10.1109/BECITHCON48839. 2019.9063197]

29. Jiang D, Ma Y, Wang Y. A multi-scale parallel convolutional neural network for automatic sleep apnea detection using single-channel EEG signals. In: Wang Y, Ma Y, Wang Y, editors. Presented at: 2018 11th International Congress on Image and Signal Processing, BioMedical Engineering and Informatics (CISP-BMEI 2018); Oct 13-15, 2018; Beijing, China. 2018.[doi: 10.1109/CISP-BMEI.2018.8633132]

30. Emin Tagluk M, Sezgin N. A new approach for estimation of obstructive sleep apnea syndrome. Expert Syst Appl. May 2011;38(5):5346-5351. [doi: 10.1016/j.eswa.2010.10.022]

31. Lin R, Lee RG, Tseng CL, Zhou HK, Chao CF, Jiang JA. A new approach for identifying sleep apnea syndrome using wavelet transform and neural networks. Biomed Eng Appl Basis Commun. Jun 25, 2006;18(3):138-143. [doi: 10.4015/ S1016237206000233]

32. Zhang Y, Li Z, Lu Y, Zhang Y, Liu G, Wang C. An EEG screening method for severe obstructive sleep apnea based on limited penetrable difference visibility graph and graph convolutional network. IEEE J Biomed Health Inform. 2025;29(11):8011-8021. [doi: 10.1109/JBHI.2025.3579556]

33. Wang Y, Ji S, Yang T, Wang X, Wang H, Zhao X. An efficient method to detect sleep hypopnea- apnea events based on EEG signals. IEEE Access. 2021;9:641-650. [doi: 10.1109/ACCESS.2020.3038486]

34. Prucnal MA, Polak AG. Analysis of features extracted from EEG epochs by discrete wavelet decomposition and Hilbert transform for sleep apnea detection. Presented at: 2018 40th Annual International Conference of the IEEE Engineering in Medicine and Biology Society (EMBC); Jul 17-21, 2018; Honolulu, HI. 2018.[doi: 10.1109/EMBC.2018.8512201]

35. Delimayanti MK, Muharram AT, Pradiptyas A, et al. Automated sleep apnea detection using CNNs: insights into the impact of FFT feature extraction on EEG signals. J Adv Inf Technol. 2025;16(9):1217-1225. [doi: 10.12720/jait.16.9. 1217-1225]

36. Zhou J, Wu XM, Zeng WJ. Automatic detection of sleep apnea based on EEG detrended fluctuation analysis and support vector machine. J Clin Monit Comput. Dec 2015;29(6):767-772. [doi: 10.1007/s10877-015-9664-0] [Medline: 25663167]

37. Saha S, Bhattacharjee A, Fattah SA. Automatic detection of sleep apnea events based on inter-band energy ratio obtained from multi-band EEG signal. Healthc Technol Lett. Jun 2019;6(3):82-86. [doi: 10.1049/htl.2018.5101] [Medline: 31341633]

38. Gupta R, Zaidi TF, Farooq O. Automatic detection of sleep apnea using sub-band features from EEG signals. In: Gupta R, Zaidi TF, editors. Presented at: 2020 3rd International Conference on Signal Processing and Information Security (ICSPIS); Nov 25-26, 2020; Dubai, United Arab Emirates. [doi: 10.1109/ICSPIS51252.2020.9340133]

39. Wang Y, Xiao Z, Fang S, Li W, Wang J, Zhao X. BI - Directional long short-term memory for automatic detection of sleep apnea events based on single channel EEG signal. Comput Biol Med. Mar 2022;142:105211. [doi: 10.1016/j. compbiomed.2022.105211]

40. Zhao X, Wang X, Yang T, et al. Classification of sleep apnea based on EEG sub-band signal characteristics. Sci Rep. 2021;11(1):5824. [doi: 10.1038/s41598-021-85138-0]

41. Bonner M, Nikolai B, Glovier Q, Lamb A, Gambhir A. Deep learning-based EEG analysis for sleep apnea detection. Presented at: 2024 Systems and Information Engineering Design Symposium (SIEDS); May 3-3, 2024; Charlottesville, VA. [doi: 10.1109/SIEDS61124.2024.10534725]

42. Gurrala V, Yarlagadda P, Koppireddi P. Detection of sleep apnea based on the analysis of sleep stages data using single channel EEG. Traitement du Signal. Apr 30, 2021;38(2):431-436. [doi: 10.18280/ts.380221]

43. Taran S, Bajaj V, Sinha GR, Polat K. Detection of sleep apnea events using electroencephalogram signals. Appl Acoust. Oct 2021;181:108137. [doi: 10.1016/j.apacoust.2021.108137]

44. Khan A, Biswas SK, Chunka C. ESAD: expert system for apnea detection using enhanced DWT feature extraction and machine learning algorithms. In: Khan A, Biswas SK, Chunka C, editors. Presented at: 2023 14th International Conference on Computing Communication and Networking Technologies (ICCCNT); Jul 6-8, 2023; Delhi, India. [doi: 10.1109/ICCCNT56998.2023.10308057]

45. Prucnal MA, Polak AG. Single-channel EEG processing for sleep apnea detection and differentiation. Metrol Meas Syst. 2023;30:323-336. [doi: 10.24425/mms.2023.144866]

46. Wijaya RS, Djamal EC, Kasyidi F. Sleep apnea identification based on EEG signals using hybrid spatio-temporal deep learning. Presented at: 2024 International Conference on Computer, Control, Informatics and Its Applications (IC3INA); Oct 9-10, 2024; Bandung, Indonesia. 2024.[doi: 10.1109/IC3INA64086.2024.10732240]

47. Bhalerao SV, Pachori RB. Sparse spectrum based swarm decomposition for robust nonstationary signal analysis with application to sleep apnea detection from EEG. Biomed Signal Process Control. Aug 2022;77:103792. [doi: 10.1016/j. bspc.2022.103792]

48. Taran S, Bajaj V, Sharma D. TEO separated AM-FM components for identification of apnea EEG signals. In: Sharma D, Bajaj V, Sharma D, editors. Presented at: 2017 IEEE 2nd International Conference on Signal and Image Processing (ICSIP); Aug 4-6, 2017; Singapore. [doi: 10.1109/SIPROCESS.2017.8124571]

49. Sharifi P, Fakharzadeh M. Algorithm for EEG—based sleep—wake classification toward sleep apnea detection. Presented at: 2025 32nd National and 10th International Iranian Conference on Biomedical Engineering (ICBME); Nov 19-20, 2025; Tabriz, Iran, Islamic Republic of. [doi: 10.1109/ICBME68496.2025.11392421]

50. Saha S, Bhattacharjee A, Fattah SA. An apnea detection method based on temporal feature variational pattern of multiband EEG signal incorporating sleep stage information. Circuits Syst Signal Process. Mar 25, 2026. [doi: 10.1007/ s00034-026-03559-6]

51. Band NC, Deshmukh C. Heuristic deep learning framework for EEG-based sleep apnea event classification. Int Res J Multidiscip Scope. 2026;07(1):1656-1665. [doi: 10.47857/irjms.2026.v07i01.08867]
