# Supplementary material for: Accuracy of Machine Learning Algorithms Based on Electroencephalogram in Sleep Apnea Detection: Systematic Review and Meta-Analysis
Source: J Med Internet Res. 2026 Jul 31;28:e93378. doi: 10.2196/93378 (PMC13427076; doi:10.2196/93378)
Supplement: Multimedia Appendix 3 [file jmir-v28-e93378-s003.docx]

Sensitivity Analysis Results


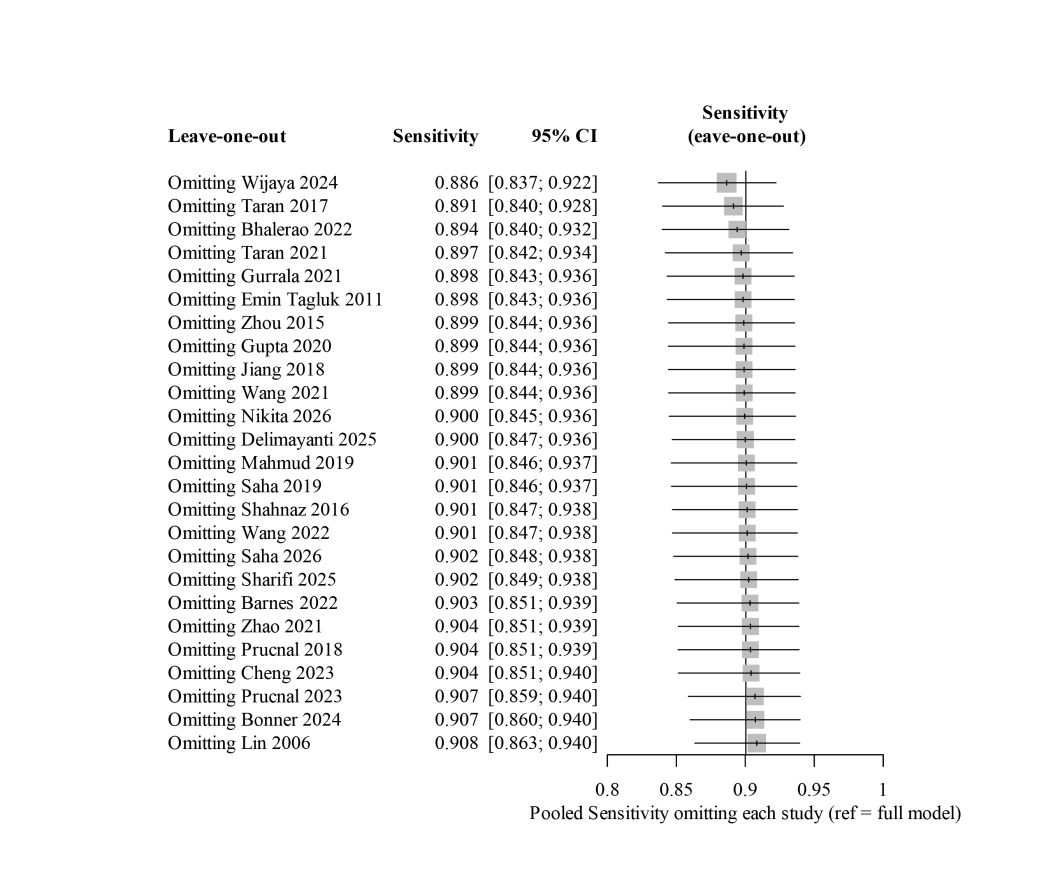


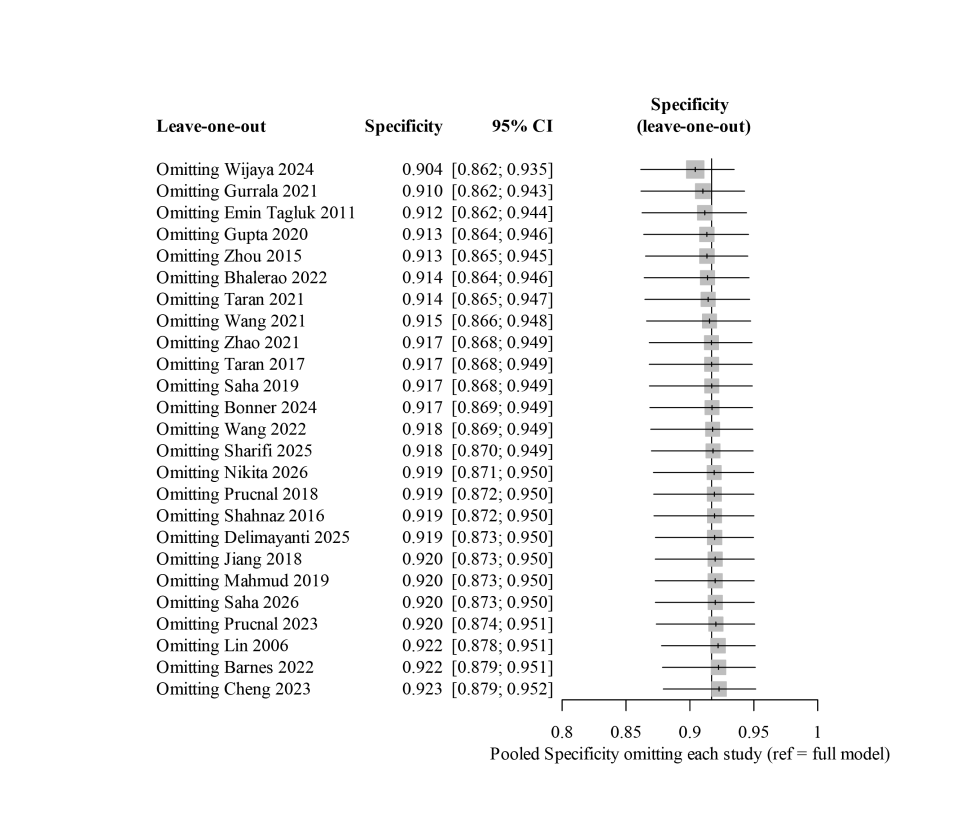


Meta Regression

| Covariate | Diagnostic parameter | Coefficient (β) | 95% CI | *P* value |
| --- | --- | --- | --- | --- |
| EEG | Sensitivity | -1.015 | -1.986 to -0.044 | .041 |
| EEG | Specificity | -1.392 | -2.330 to -0.454 | .004 |
| Classifer | Sensitivity | -0.157 | -1.077 to 0.763 | .738 |
| Classifer | Specificity | 0.335 | -0.615 to 1.284 | .490 |
| Feature extraction | Sensitivity | -0.339 | -1.202 to 0.524 | .441 |
| Feature extraction | Specificity | 0.038 | -1.014 to 1.090 | .944 |
| Region | Sensitivity | 1.564 | 0.533 to 2.594 | .003 |
| Region | Specificity | 0.965 | -0.201 to 2.131 | .105 |
| Database | Sensitivity | 0.404 | -0.877 to 1.685 | .536 |
| Database | Specificity | 0.110 | -1.188 to 1.409 | .868 |
| Validation method | Sensitivity | -0.104 | -1.127 to 0.919 | .842 |
| Validation method | Specificity | -1.163 | -2.303 to -0.022 | .046 |

### References

19. Shahnaz C, Minhaz AT, Ahamed S. Sub-frame based apnea detection exploiting delta band power ratio extracted from EEG signals. In: Shahnaz C, Minhaz AT, Ahamed ST, editors. Presented at: TENCON 2016—2016 IEEE Region 10 Conference; Nov 22-25, 2016; Singapore. 2016.[doi: 10.1109/TENCON.2016.7847987]

26. Cheng L, Luo S, Li B, Liu R, Zhang Y, Zhang H. Multiple-instance learning for EEG based OSA event detection. Biomed Signal Process Control. Feb 2023;80:104358. [doi: 10.1016/j.bspc.2022.104358]

27. Barnes LD, Lee K, Kempa-Liehr AW, Hallum LE. Detection of sleep apnea from single-channel electroencephalogram (EEG) using an explainable convolutional neural network (CNN). PLoS One. 2022;17(9):e0272167. [doi: 10.1371/ journal.pone.0272167] [Medline: 36099242]

28. Mahmud T, Aeioub Ansary M, Mahmud TI, Khan IA, Fattah SA. Real time sleep apnea event detection with deep neural network. Presented at: 2019 IEEE International Conference on Biomedical Engineering, Computer and Information Technology for Health (BECITHCON); Nov 28-30, 2019; Dhaka, Bangladesh. 2019.[doi: 10.1109/BECITHCON48839. 2019.9063197]

29. Jiang D, Ma Y, Wang Y. A multi-scale parallel convolutional neural network for automatic sleep apnea detection using single-channel EEG signals. In: Wang Y, Ma Y, Wang Y, editors. Presented at: 2018 11th International Congress on Image and Signal Processing, BioMedical Engineering and Informatics (CISP-BMEI 2018); Oct 13-15, 2018; Beijing, China. 2018.[doi: 10.1109/CISP-BMEI.2018.8633132]

30. Emin Tagluk M, Sezgin N. A new approach for estimation of obstructive sleep apnea syndrome. Expert Syst Appl. May 2011;38(5):5346-5351. [doi: 10.1016/j.eswa.2010.10.022]

31. Lin R, Lee RG, Tseng CL, Zhou HK, Chao CF, Jiang JA. A new approach for identifying sleep apnea syndrome using wavelet transform and neural networks. Biomed Eng Appl Basis Commun. Jun 25, 2006;18(3):138-143. [doi: 10.4015/ S1016237206000233]

33. Wang Y, Ji S, Yang T, Wang X, Wang H, Zhao X. An efficient method to detect sleep hypopnea- apnea events based on EEG signals. IEEE Access. 2021;9:641-650. [doi: 10.1109/ACCESS.2020.3038486]

34. Prucnal MA, Polak AG. Analysis of features extracted from EEG epochs by discrete wavelet decomposition and Hilbert transform for sleep apnea detection. Presented at: 2018 40th Annual International Conference of the IEEE Engineering in Medicine and Biology Society (EMBC); Jul 17-21, 2018; Honolulu, HI. 2018.[doi: 10.1109/EMBC.2018.8512201]

35. Delimayanti MK, Muharram AT, Pradiptyas A, et al. Automated sleep apnea detection using CNNs: insights into the impact of FFT feature extraction on EEG signals. J Adv Inf Technol. 2025;16(9):1217-1225. [doi: 10.12720/jait.16.9. 1217-1225]

36. Zhou J, Wu XM, Zeng WJ. Automatic detection of sleep apnea based on EEG detrended fluctuation analysis and support vector machine. J Clin Monit Comput. Dec 2015;29(6):767-772. [doi: 10.1007/s10877-015-9664-0] [Medline: 25663167]

37. Saha S, Bhattacharjee A, Fattah SA. Automatic detection of sleep apnea events based on inter-band energy ratio obtained from multi-band EEG signal. Healthc Technol Lett. Jun 2019;6(3):82-86. [doi: 10.1049/htl.2018.5101] [Medline: 31341633]

38. Gupta R, Zaidi TF, Farooq O. Automatic detection of sleep apnea using sub-band features from EEG signals. In: Gupta R, Zaidi TF, editors. Presented at: 2020 3rd International Conference on Signal Processing and Information Security (ICSPIS); Nov 25-26, 2020; Dubai, United Arab Emirates. [doi: 10.1109/ICSPIS51252.2020.9340133]

39. Wang Y, Xiao Z, Fang S, Li W, Wang J, Zhao X. BI - Directional long short-term memory for automatic detection of sleep apnea events based on single channel EEG signal. Comput Biol Med. Mar 2022;142:105211. [doi: 10.1016/j. compbiomed.2022.105211]

40. Zhao X, Wang X, Yang T, et al. Classification of sleep apnea based on EEG sub-band signal characteristics. Sci Rep. 2021;11(1):5824. [doi: 10.1038/s41598-021-85138-0]

41. Bonner M, Nikolai B, Glovier Q, Lamb A, Gambhir A. Deep learning-based EEG analysis for sleep apnea detection. Presented at: 2024 Systems and Information Engineering Design Symposium (SIEDS); May 3-3, 2024; Charlottesville, VA. [doi: 10.1109/SIEDS61124.2024.10534725]

42. Gurrala V, Yarlagadda P, Koppireddi P. Detection of sleep apnea based on the analysis of sleep stages data using single channel EEG. Traitement du Signal. Apr 30, 2021;38(2):431-436. [doi: 10.18280/ts.380221]

43. Taran S, Bajaj V, Sinha GR, Polat K. Detection of sleep apnea events using electroencephalogram signals. Appl Acoust. Oct 2021;181:108137. [doi: 10.1016/j.apacoust.2021.108137]

45. Prucnal MA, Polak AG. Single-channel EEG processing for sleep apnea detection and differentiation. Metrol Meas Syst. 2023;30:323-336. [doi: 10.24425/mms.2023.144866]

46. Wijaya RS, Djamal EC, Kasyidi F. Sleep apnea identification based on EEG signals using hybrid spatio-temporal deep learning. Presented at: 2024 International Conference on Computer, Control, Informatics and Its Applications (IC3INA); Oct 9-10, 2024; Bandung, Indonesia. 2024.[doi: 10.1109/IC3INA64086.2024.10732240]

47. Bhalerao SV, Pachori RB. Sparse spectrum based swarm decomposition for robust nonstationary signal analysis with application to sleep apnea detection from EEG. Biomed Signal Process Control. Aug 2022;77:103792. [doi: 10.1016/j. bspc.2022.103792]

48. Taran S, Bajaj V, Sharma D. TEO separated AM-FM components for identification of apnea EEG signals. In: Sharma D, Bajaj V, Sharma D, editors. Presented at: 2017 IEEE 2nd International Conference on Signal and Image Processing (ICSIP); Aug 4-6, 2017; Singapore. [doi: 10.1109/SIPROCESS.2017.8124571]

49. Sharifi P, Fakharzadeh M. Algorithm for EEG—based sleep—wake classification toward sleep apnea detection. Presented at: 2025 32nd National and 10th International Iranian Conference on Biomedical Engineering (ICBME); Nov 19-20, 2025; Tabriz, Iran, Islamic Republic of. [doi: 10.1109/ICBME68496.2025.11392421]

50. Saha S, Bhattacharjee A, Fattah SA. An apnea detection method based on temporal feature variational pattern of multiband EEG signal incorporating sleep stage information. Circuits Syst Signal Process. Mar 25, 2026. [doi: 10.1007/ s00034-026-03559-6]

51. Band NC, Deshmukh C. Heuristic deep learning framework for EEG-based sleep apnea event classification. Int Res J Multidiscip Scope. 2026;07(1):1656-1665. [doi: 10.47857/irjms.2026.v07i01.08867]
