# Supplementary material for: Accuracy of Machine Learning Algorithms Based on Electroencephalogram in Sleep Apnea Detection: Systematic Review and Meta-Analysis
Source: J Med Internet Res. 2026 Jul 31;28:e93378. doi: 10.2196/93378 (PMC13427076; doi:10.2196/93378)
Supplement: Multimedia Appendix 4 [file jmir-v28-e93378-s004.doc]

**User：**

Improve it

Currently, polysomnography (PSG) is considered the gold standard for the diagnosis of sleep apnea (SA). PSG records multiple physiological parameters during overnight sleep, including electroencephalogram (EEG), electrocardiogram (ECG), and blood oxygen saturation, in a sleep laboratory environment. However, PSG has several limitations, including high cost, long waiting times, and the requirement for specialized facilities and trained personnel, which limit its widespread clinical application[1]. Moreover, the manual analysis of PSG recordings is time-consuming and labor-intensive, and the results may be influenced by subjective interpretation bias[2]. Therefore, developing more convenient and efficient auxiliary diagnostic methods is of great clinical significance.

**ChatGPT：**

Currently, polysomnography (PSG) serves as the gold standard for diagnosing sleep apnea (SA). This assessment involves overnight recording of multiple physiological signals—including electroencephalogram (EEG), electrocardiogram (ECG), and blood oxygen saturation—in a laboratory setting. However, PSG faces limitations such as high costs, lengthy wait times, and dependence on specialized sleep laboratories, which restrict its widespread application[1]. Additionally, manual interpretation of PSG data is time-consuming, labor-intensive, and prone to subjective bias[2]. Therefore, the pursuit of more convenient and efficient auxiliary diagnostic tools holds significant clinical importance.

**User：**

Improve it

Electroencephalogram (EEG), as a non-invasive technology, records the electrical activity of the brain through electrodes placed on the scalp surface[3]. Although EEG has some limitations, such as the need to attach electrodes, the use of conductive gel, and discomfort caused by long-term monitoring, compared with other physiological signals such as electrocardiogram and respiratory airflow, EEG is less affected by endogenous physiological factors, including irregular breathing and arrhythmia, during sleep monitoring. EEG can directly reflect neuronal activity and sleep states, providing more direct and abundant physiological information for the evaluation of sleep apnea[4].

**ChatGPT：**

Electroencephalogram (EEG), as a non-invasive technique, records the brain's electrical activity by placing electrodes on the scalp surface[3]. Although EEG has limitations such as the application of electrodes, the use of conductive gel, and potential discomfort associated with prolonged monitoring, but compared to other physiological signals such as electrocardiograms and respiratory airflow, EEG is less susceptible to interference from endogenous physiological factors like irregular breathing and arrhythmia when used for sleep monitoring. It directly reflects neuronal activity and sleep states in the brain, providing richer and more direct physiological insights for the assessment of sleep apnea[4]Error: Reference source not found.

**User：**

Improve it

Studies were divided into segment-level and patient-level studies according to the unit of analysis. Segment-level studies usually divide EEG signals into fixed-length time windows (such as 30-second epochs or 10-second segments), and each segment is regarded as an independent sample for feature extraction and classification[5]. Patient-level studies combine EEG features collected throughout the whole night and provide one diagnostic outcome for each patient. To ensure the independence of samples, studies based on sub-frame analysis generally use non-overlapping fixed-length segmentation methods. However, sleep apnea events may occur across the boundaries of sub-frames. Therefore, some studies apply overlapping sliding windows and aggregate sub-frame features at the feature level to obtain a global feature representation, which can preserve the integrity of apnea events while reducing the dependence between samples[6]. Regarding data splitting, patient-level division of training and testing datasets (all data from one patient are assigned entirely to either the training or testing set) can effectively avoid data leakage and improve model generalization. In contrast, when EEG segments from the same patient are randomly divided into both training and testing sets, information leakage may occur, resulting in an overestimation of model performance[7].

**ChatGPT：**

Studies are categorized into segment-level and patient-level designs based on the unit of analysis. Segment-level studies analyze EEG data using fixed time windows (e.g., 30-second epochs, 10-second frames), treating each segment as an independent unit for feature extraction and classification[5]. Patient-level studies aggregate features recorded throughout the night to generate a single diagnostic result for each patient. To maintain sample independence, studies based on sub-frame analysis typically employ non-overlapping, fixed-length segmentation. However, sleep apnea events may span sub-frame boundaries; some studies use overlapping sliding windows and perform feature aggregation of sub-frame features at the feature level to generate a single global feature, thereby preserving event integrity while reducing statistical dependencies between samples[6]. At the data partitioning level, the patient-based training-test set division (where all data from a single participant is fully allocated to either the training set or the test set) effectively prevents data leakage and ensures model generalization. Conversely, if EEG segments from the same participant are distributed across both the training and test sets, this may lead to severe information leakage and an overestimation of performance[7].

**User：**

Improve it

The results of QUADAS-2 assessment showed that, in terms of patient selection, 2 studies (7.4%) were considered to have a high risk of bias because of inappropriate participant selection and artificial balancing of samples between groups. In addition, 10 studies (37.0%) did not provide sufficient information regarding the participant selection process, and their risk of bias was considered unclear. The remaining 15 studies (55.6%) were considered to have a low risk of bias. Regarding flow and timing, 5 studies (18.5%) had unclear reporting of the interval between the index test and the reference standard, resulting in an unclear risk of bias. The other domains were all judged as having a low risk of bias.

**ChatGPT：**

The QUADAS-2 assessment results showed that, in the area of patient selection, 2 studies (7.4%) were judged to be at high risk of bias due to inappropriate subject selection and artificial balancing of groups. Another 10 studies (37.0%) did not adequately describe the subject selection process, and their risk of bias was judged to be unclear; The remaining 15 studies (55.6%) were classified as having a low risk of bias. In the areas of flow and timing, the interval between the test under evaluation and the reference standard was unclear in 5 studies (18.5%), and the risk of bias was judged to be unclear. All other areas were classified as having a low risk of bias.

**User：**

Improve it

A segment-level meta-analysis was conducted including 27 studies with 32 sets of four-cell table data. Spearman correlation analysis showed a significant correlation between the logit of sensitivity and the logit of (1-specificity) (r=-0.685, P<.001), suggesting the existence of a threshold effect among the included studies. Therefore, the heterogeneity may mainly come from different diagnostic thresholds. Based on this result, a bivariate random-effects model was applied to generate the summary receiver operating characteristic (SROC) curve. The pooled area under the curve (AUC) was 0.95 (95% CI 0.92-0.99; Figure 6), indicating that EEG-based machine learning models had good diagnostic performance for sleep apnea detection. The pooled sensitivity was 0.90 (95% CI 0.85-0.94; I²=99.5%; Figure 4), and the pooled specificity was 0.92 (95% CI 0.87-0.95; I²=99.8%; Figure 5).

**ChatGPT：**

A segment-level meta-analysis included 27 studies comprising 32 sets of four-cell table data. Spearman correlation analysis demonstrated a significant correlation between logit-transformed sensitivity and logit(1−specificity) (r=−0.685, P<.001), indicating a pronounced threshold effect among the included studies and suggesting that between-study heterogeneity was primarily attributable to differences in classification thresholds. Therefore, a bivariate random-effects model was used to construct the summary receiver operating characteristic (SROC) curve. The pooled area under the curve (AUC) was 0.95 (95% CI 0.92-0.99; Figure 6), showing a promising overall diagnostic performance of EEG-based machine learning models for sleep apnea detection. The pooled sensitivity was 0.90 (95% CI 0.85-0.94; I²=99.5%; Figure 4), and the pooled specificity was 0.92 (95% CI 0.87-0.95; I²=99.8%; Figure 5).

**User：**

Improve it

Although the pooled results showed high diagnostic accuracy for detecting both positive and negative sleep apnea cases, significant heterogeneity existed among the included studies, indicating that the performance of machine learning models varied across different studies. In addition, the 95% prediction intervals of sensitivity (0.43-0.99) and specificity (0.46-0.99) were relatively wide, suggesting that the diagnostic performance of these models may be different when applied to external validation datasets or real-world clinical environments, which may affect their stability and generalization ability.

**ChatGPT：**

Although the pooled estimates suggested high diagnostic accuracy for both positive and negative sleep apnea cases, substantial between-study heterogeneity was observed, indicating considerable variability in model performance across different study settings. Furthermore, the 95% prediction intervals were wide for both sensitivity (0.43-0.99) and specificity (0.46-0.99), suggesting that diagnostic performance may vary substantially in external validation scenarios or future real-world applications, thereby reflecting limited model stability and generalizability.

**User：**

Improve it

Meta-regression analysis showed that EEG electrode configuration was significantly related to pooled sensitivity and specificity. Regional factors and validation methods were associated with sensitivity and specificity, respectively. These results indicated that differences in EEG electrode configuration, study regions, and validation strategies might be the potential sources of heterogeneity among the included studies. Subgroup analysis found that multi-channel EEG models had better diagnostic performance than single-channel EEG models (sensitivity: 0.94 vs 0.87; specificity: 0.92 vs 0.90). However, the differences between groups were not statistically significant, and the results should be interpreted carefully. Studies from Asian populations showed slightly higher diagnostic performance compared with studies from Western populations, and hold-out validation methods showed better performance than cross-validation methods. However, these results might be affected by the limited number of studies included in each subgroup. The risk of bias among included studies was low to moderate, and the GRADE assessment showed that the overall certainty of evidence was low, mainly due to substantial heterogeneity and risk of bias in some studies. Therefore, although the pooled diagnostic performance of EEG-based machine learning models appeared promising, the reliability and generalizability of the findings remain uncertain.

**ChatGPT：**

Meta-regression analysis showed that EEG electrode configuration was significantly associated with both pooled sensitivity and specificity, whereas regional factors and validation strategies were associated with sensitivity and specificity, respectively. These findings suggest that differences in electrode configuration, regional background, and validation methods across studies may represent potential sources of heterogeneity. Subgroup analysis further showed that the multi-lead EEG model outperformed the single-lead model (sensitivity: 0.94 vs. 0.87; specificity: 0.92 vs. 0.90). However, the between-group differences did not reach statistical significance, and these findings should therefore be interpreted with caution. Studies conducted in Asian populations showed slightly better diagnostic performance than those in Western populations, and hold-out validation appeared to outperform cross-validation; however, these findings may be influenced by small subgroup sample sizes. However, the risk of bias in the included studies was low to moderate, and following a GRADE assessment, the overall certainty of the evidence was judged to be low, primarily due to high heterogeneity and risk of bias in some studies. Therefore, although the pooled estimates suggest promising diagnostic performance, confidence in the magnitude and generalizability of these estimates remains limited.

**User：**

Improve it

In addition, sleep stage information has an important influence on the performance of EEG-based sleep apnea detection models. The combined sleep stage and OSA detection model proposed by Kang et al. [8] showed that, in younger subjects, EEG features including K-complexes, sleep spindles, and beta-band activity were significantly different between REM and NREM sleep stages, while in older subjects, these differences were only found during NREM sleep. These results indicate that the relationship between sleep stages and EEG features may vary with age. Therefore, models that do not consider sleep stage information may have inconsistent diagnostic performance in different populations.

**ChatGPT：**

In addition, sleep stage information has a significant impact on the performance of EEG-based sleep apnea detection models. The combined sleep stage and OSA screening model developed by Kang et al. [8] found that in younger individuals, EEG features (K-complexes, spindles, and beta-band activity) differed significantly between REM and NREM sleep, whereas in older adults, these differences were only observed during NREM sleep. This suggests that the interaction between sleep stages and EEG features varies by age, and models that fail to account for sleep stage information may exhibit inconsistent diagnostic performance across populations.

**User：**

Improve it

Despite the good performance at the segment level in this meta-analysis, how to translate segment-level prediction results into clinically meaningful patient-level diagnosis remains a challenge for EEG-based machine learning models. In clinical practice, the severity of sleep apnea is mainly assessed by the apnea-hypopnea index (AHI), which is calculated according to the number of apnea and hypopnea events per hour of sleep[9]. Therefore, accurate diagnosis at the patient level requires not only the detection of respiratory events during the whole sleep period but also the accurate calculation of total sleep time and the integration of detected events into clinically meaningful AHI values. However, most of the included studies mainly focused on the classification performance of EEG segments and did not assess whether segment-level predictions could be converted into reliable patient-level AHI estimation. In addition, current EEG-based studies mainly focused on apnea detection and did not clearly identify hypopnea events, which may further restrict their clinical application.

**ChatGPT：**

Despite the favorable segment-level performance observed in this meta-analysis, translating segment-level prediction into clinically meaningful patient-level diagnosis remains a major challenge for EEG-based machine learning models. In clinical practice, sleep apnea severity is primarily determined using the apnea-hypopnea index (AHI), which is calculated based on the total number of apnea and hypopnea events per hour of sleep[9]. Therefore, accurate patient-level diagnosis requires not only reliable detection of respiratory events throughout the entire sleep recording but also accurate estimation of total sleep time and aggregation of detected events into clinically interpretable AHI values. However, many included studies focused primarily on segment-level classification performance and did not evaluate whether segment-level predictions could be reliably translated into patient-level AHI estimation. Furthermore, the included EEG-based studies primarily focus on apnea detection and do not clearly distinguish hypopnea events, which may further limit clinical applicability.

**User：**

Improve it

Future studies should focus on establishing standardized criteria for model evaluation, threshold determination, and result reporting to improve the consistency and comparability among different studies. Although multi-channel EEG models showed better diagnostic performance, future research should further explore appropriate channel configurations according to different clinical applications and real-world environments. In addition, AI-based EEG screening systems need more prospective validation in real-world clinical settings, especially regarding clinical effectiveness, feasibility, and economic benefits. Emerging approaches such as federated learning may provide new opportunities for multi-center data sharing and model development while protecting patient privacy, thereby improving the stability and generalization ability of models across different regions, populations, and healthcare systems[10]. Meanwhile, explainable artificial intelligence methods (such as SHAP, LIME, and Grad-CAM) may help overcome the “black-box” problem of deep learning models and enhance model interpretability and clinicians’ confidence in AI-assisted decision-making[11]. Finally, continuous external validation, bias assessment, and model updating are important to ensure the fairness, robustness, and long-term reliability of AI models in clinical practice[12].

**ChatGPT：**

Future research should prioritize establishing a standardized framework for model evaluation, threshold selection, and result reporting to improve consistency and comparability across studies. While leveraging the diagnostic advantages of multi-channel EEG, future studies should also develop channel configuration strategies tailored to different clinical and real-world scenarios. In addition, AI-assisted EEG screening systems require further prospective validation in real-world settings, with emphasis on clinical utility, feasibility, and cost-effectiveness. Novel collaborative frameworks such as federated learning may facilitate multi-center data sharing and model training while preserving patient privacy, thereby improving model stability and generalizability across regions, populations, and healthcare systems[10]. In parallel, explainable AI methods (e.g., SHAP, LIME, and Grad-CAM) may help address the “black-box” limitation of deep learning models, improving interpretability and clinician trust in AI-assisted decision-making[11]. Finally, continued external validation, bias monitoring, and dynamic model updating remain essential to ensure fairness, stability, and long-term reliability in clinical practice[12].

1. AlGhanim N, Comondore VR, Fleetham J, Marra CA, Ayas NT. The economic impact of obstructive sleep apnea. Lung. 2008 2008;186(1):7-12.

2. Alberto Nigro C, Malnis S, Dibur E, Rhodius E. How reliable is the manual correction of the autoscoring of a level IV sleep study (ApneaLink™) by an observer without experience in polysomnography? Sleep & breathing. 2012 2012;16(2):275-9.

3. Ji Z, Li L, Zheng M, Ye X, Yan W, Wang Z, et al. Conductive Hydrogel‐Enabled Electrode for Scalp Electroencephalography Monitoring. Small Methods. 2025 2025-09-06:e01242. doi: 10.1002/smtd.202501242.

4. Fathima S, Ahmed M. Sleep Apnea Detection Using EEG: A Systematic Review of Datasets, Methods, Challenges, and Future Directions. Annals of Biomedical Engineering. 2025 05/2025;53(5):1043-67. doi: 10.1007/s10439-025-03691-5.

5. Zhang DH, Zhou J, Wickens JD, Veale AG, Hallum LE. APNEA AND HYPOPNEA EVENT DETECTION USING EEG, EMG, AND SLEEP STAGE LABELS IN A COHORT OF PATIENTS WITH SUSPECTED SLEEP APNEA. medRxiv. 2024 2024((Zhang D.H.; Zhou J.; Wickens J.D.; Hallum L.E., l.hallum@auckland.ac.nz) Faculty of Engineering, University of Auckland, 20 Symonds Street, Auckland Central, Auckland, New Zealand). doi: 10.1101/2024.10.24.24316077.

6. Shahnaz C, Minhaz AT, Ahamed ST, editors. Sub-frame based apnea detection exploiting delta band power ratio extracted from EEG signals. TENCON 2016 - 2016 IEEE Region 10 Conference; 2016 11/2016. Singapore: IEEE.

7. Shamsi H. Alzheimer’s diagnosis from EEG with reliable probabilities: subject-wise, leakage-free evaluation and isotonic calibration. Journal of Engineering and Applied Science. 2025 12/2025;72(1):226. doi: 10.1186/s44147-025-00821-7.

8. Kang C, An S, Kim HJ, Devi M, Cho A, Hwang S, et al. Age-integrated artificial intelligence framework for sleep stage classification and obstructive sleep apnea screening. Frontiers in Neuroscience. 2023 2023;17((Kang C.; Devi M.; Lee H.W., leeh@ewha.ac.kr) Computational Medicine, System Health Science and Engineering Program, Ewha Womans University, Seoul, South Korea). doi: 10.3389/fnins.2023.1059186.

9. Berry RB, Budhiraja R, Gottlieb DJ, Gozal D, Iber C, Kapur VK, et al. Rules for Scoring Respiratory Events in Sleep: Update of the 2007 AASM Manual for the Scoring of Sleep and Associated Events: Deliberations of the Sleep Apnea Definitions Task Force of the American Academy of Sleep Medicine. Journal of Clinical Sleep Medicine. 2012 2012-10-15;8(5):597-619. doi: 10.5664/jcsm.2172.

10. Rajpurkar P, Chen E, Banerjee O, Topol EJ. AI in health and medicine. Nat Med. 2022 Jan;28(1):31-8. PMID: 35058619. doi: 10.1038/s41591-021-01614-0.

11. S Band S, Yarahmadi A, Hsu C-C, Biyari M, Sookhak M, Ameri R, et al. Application of explainable artificial intelligence in medical health: A systematic review of interpretability methods. Informatics in Medicine Unlocked. 2023 2023;40:101286. doi: 10.1016/j.imu.2023.101286.

12. Davis SE, Greevy RA, Jr., Lasko TA, Walsh CG, Matheny ME. Detection of calibration drift in clinical prediction models to inform model updating. J Biomed Inform. 2020 Dec;112:103611. PMID: 33157313. doi: 10.1016/j.jbi.2020.103611.
